# Structural mechanism for bidirectional actin cross-linking by T-plastin

**DOI:** 10.1073/pnas.2205370119

**Published:** 2022-09-06

**Authors:** Lin Mei, Matthew J. Reynolds, Damien Garbett, Rui Gong, Tobias Meyer, Gregory M. Alushin

**Affiliations:** ^a^Laboratory of Structural Biophysics and Mechanobiology, The Rockefeller University, New York, NY 10065;; ^b^Tri-Institutional PhD Program in Chemical Biology, The Rockefeller University, New York, NY 10065;; ^c^Department of Chemical and Systems Biology, Stanford University School of Medicine, Stanford, CA 94305;; ^d^Department of Cell and Developmental Biology, Weill Cornell Medical College, New York, NY 10065

**Keywords:** actin cytoskeleton, cryo-EM, plastin, fimbrin

## Abstract

To orchestrate cell mechanics, trafficking, and motility, cytoskeletal filaments must assemble into higher-order networks whose local subcellular architecture and composition specify their functions. Cross-linking proteins bridge filaments at the nanoscale to control a network’s μm-scale geometry, thereby conferring its mechanical properties and functional dynamics. While these interfilament linkages are key determinants of cytoskeletal function, their structural mechanisms remain poorly understood. Plastins/fimbrins are an evolutionarily ancient family of tandem calponin-homology domain (CHD) proteins required to construct multiple classes of actin networks, which feature diverse geometries specialized to power cytokinesis, microvilli and stereocilia biogenesis, and persistent cell migration. Here, we focus on the structural basis of actin network assembly by human T-plastin, a ubiquitously expressed isoform necessary for the maintenance of stable cellular protrusions generated by actin polymerization forces. By implementing a machine-learning–enabled cryo-electron microscopy pipeline for visualizing cross-linkers bridging multiple filaments, we uncover a sequential bundling mechanism enabling T-plastin to bridge pairs of actin filaments in both parallel and antiparallel orientations. T-plastin populates distinct structural landscapes in these two bridging orientations that are selectively compatible with actin networks featuring divergent architectures and functions. Our structural, biochemical, and cell biological data highlight inter-CHD linkers as key structural elements underlying flexible but stable cross-linking that are likely to be disrupted by T-plastin mutations that cause hereditary bone diseases.

## Significance

To fulfill the cytoskeleton’s diverse functions in cell mechanics and motility, actin networks with specialized architectures are built by cross-linking proteins. How these cross-linkers specify cytoskeletal network geometry is poorly understood at the level of protein structure. Here, we introduce a machine-learning–enabled pipeline for visualizing cross-linkers bridging cytoskeletal filaments with cryogenic electron microscopy (cryo-EM). We apply our method to T-plastin, a member of the evolutionarily conserved plastin/fimbrin family, revealing a sequence of conformational changes that enables T-plastin to bridge pairs of actin filaments in both parallel and antiparallel orientations. This provides a structural framework for understanding how plastins can generate actin networks featuring mixed filament polarity.

Actin filaments (F-actin) must be incorporated into μm-scale higher-order assemblies along with dozens of actin-binding proteins (ABPs) at specific subcellular locations for the cytoskeleton to fulfill its functions (1–3). Branched F-actin, classically associated with the plasma membrane to propel cell migration, has been extensively characterized through structural studies of the ARP2/3 complex (4), the sole branched-actin nucleator, culminating in recent in situ subtomogram averaging (5) and in vitro single-particle cryo-electron microscopy (cryo-EM) structures (6). The other major class of actin assemblies, F-actin bundles composed primarily of collinear filaments, remains broadly poorly understood at the protein structural level. In contrast to the central role of ARP2/3 in forming branched F-actin, F-actin bundles are compositionally and functionally diverse. They underlie the acrosome of sperm (7); tubular membrane protrusions including filopodia (8), microvilli (9), and stereocilia (10); and contractile networks including muscle fibers (11, 12), stress fibers (13), and the cytokinetic ring (1). Each type of F-actin bundle network features distinctive cross-linkers that specify its nanoscale architecture (relative filament polarities, spacings, and orientations), thereby controlling which ABPs and myosin motor proteins locally engage a network to confer its specific mechanical properties and biochemical activities. Pioneering studies of paracrystalline acrosomal bundles (7, 14, 15) and in vitro assembled two-dimensional (2D) actin arrays (16–20), as well as recent subtomogram averaging studies of muscle fibers (21, 22), have provided foundational insights into how the domain organization of cross-linkers is linked to network architectures, yet detailed mechanisms remain obscure. Despite recent progress in cryo-EM analysis of individual actin filaments in complex with ABPs (23), including fragments of actin-bundling proteins (24–28), to our knowledge no high-resolution structures of full-length cross-linkers bridging cytoskeletal filaments have been reported, perpetuating a major gap in understanding their mechanisms.

Here we focus on human T-plastin, a member of the plastin/fimbrin family of tandem calponin-homology domain (CHD) proteins. Plastins are highly conserved throughout eukaryotes, suggesting that they fulfill an ancient function in actin bundling (29, 30). Consistently, a plastin/spectrin double knockout in the *Caenorhabditis elegans* embryo has recently been reported to result in failure of cytokinesis (31), likely the most ancestral function of the actin cytoskeleton (32). Humans feature three highly similar plastin isoforms: I-plastin (PLS1/fimbrin), expressed in the kidney, the intestine, and the inner ear, where it localizes to microvilli and stereocilia (33, 34); L-plastin (PLS2/LCP1), natively expressed in leukocytes (35) and ectopically expressed in many cancers (36); and T-plastin (PLS3), the most abundant isoform, which is ubiquitously expressed in solid tissues (37) and dysregulated in cancers (38). T-plastin is a 70.8 kDa monomeric protein that functions as a key actin network stabilizer, strengthening and promoting cell protrusions by localizing to lamellipodia and filopodia (8). It is involved in many cellular processes requiring dynamic cytoskeletal reorganization including cell migration (8), endocytosis (39), and membrane remodeling (40). T-plastin is associated with autosomal recessive spinal muscular atrophy (41), and mutations in T-plastin have recently been reported to cause congenital osteoporosis (27, 42–45).

**Fig. 1.**
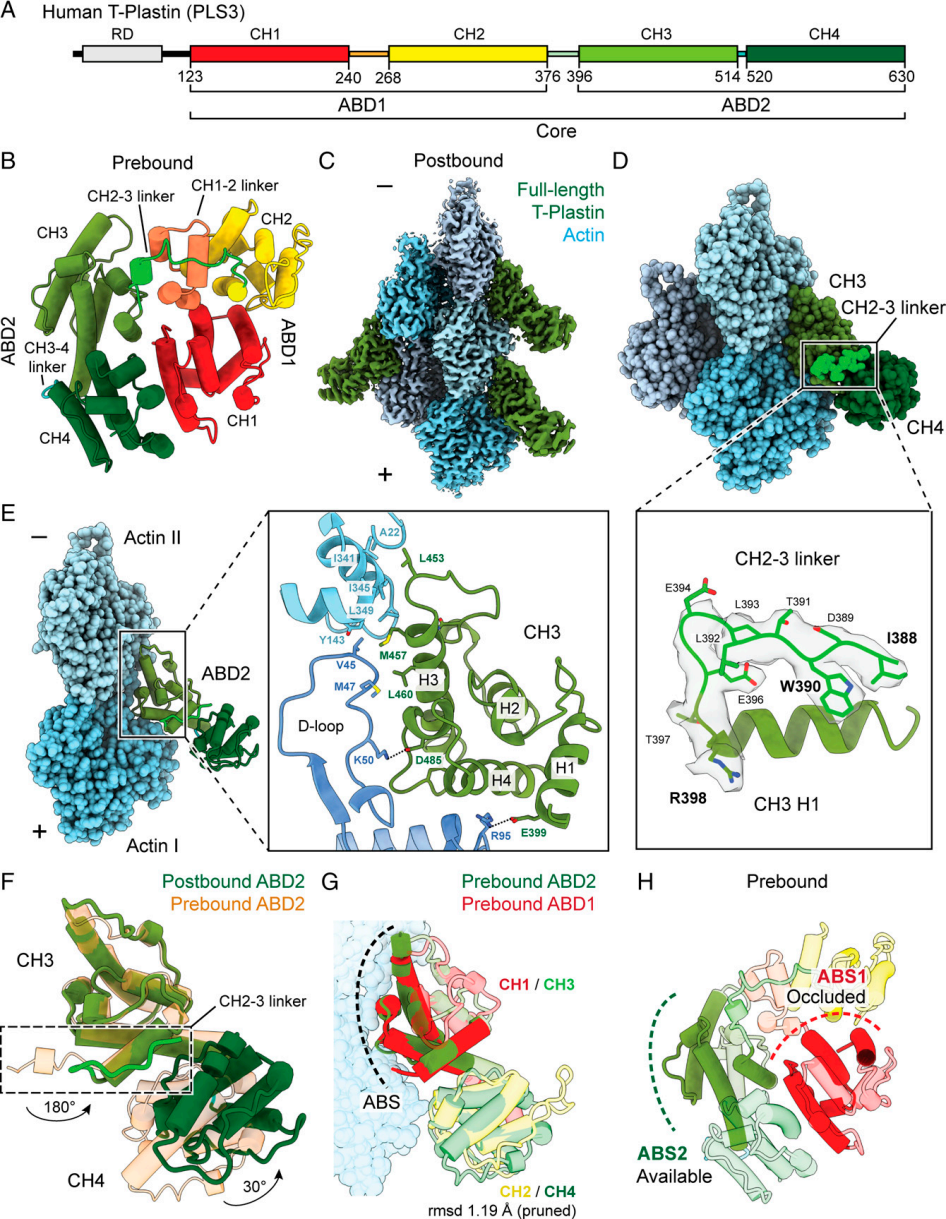
Cryo-EM structure of full-length T-plastin bound to single actin filaments resolves ABD2. A cryo-EM structure in the presence of Ca^2+^ indicates that T-plastin’s ABD2 engages F-actin first. (*A*) Domain structure of human T-plastin. Primary sequence boundaries of CHDs are indicated. (*B*) Prebound homology model of T-plastin’s actin-binding core. (*C*) Segmented region of the postbound T-plastin–decorated–F-actin cryo-EM map (2.6 Å resolution) in the presence of Ca^2+^. (*D*) Postbound T-plastin–F-actin complex atomic model. Actin subunits are displayed in varying shades of blue. CH3: olive; CH4: dark green. The inter-ABD CH2–3 linker, highlighted in bright green, is displayed along with its segmented density in the lower box. (*E*) The actin-binding interface of ABD2. (*F*) Superimposed prebound and postbound models of ABD2. The conformational change of the CH2–3 linker is highlighted (box). Rotation angles indicate repositioning of the CH2–3 linker as well as CH4 relative to CH3. (*G*) Superimposed ABD1 and ABD2 from prebound model on postbound ABD2 (not shown). ABS, actin-binding site. Actin from the postbound model is displayed. (*H*) Actin-binding sites are highlighted on the prebound model.

While most cross-linkers bridge filaments through the dimerization or tetramerization of subunits featuring a single actin-binding domain (ABD) (46), plastins contain two ABDs (ABD1 and ABD2) within a single polypeptide chain, each composed of two tandem CHDs (Fig. 1*A*). They also feature a flexibly tethered N-terminal regulatory domain (RD) consisting of two Ca^2+^-binding EF-hand motifs (Fig. 1*A*), which inhibits bundling activity in the presence of Ca^2+^ while still licensing binding to single actin filaments by unclear mechanisms (47, 48). When purified, fission yeast fimbrin can promote the formation of both parallel and antiparallel F-actin bundles (39), suggesting that individual plastin molecules must possess the capacity to bridge actin filaments in radically different geometries. The actin-binding cores (lacking the flexible RD) of *Schizosaccharomyces pombe* and *Arabidopsis thaliana* fimbrin have been crystallized (49) in the absence of actin, and the isolated ABD2 of L-plastin (27, 50) and ABD1 of T-plastin (51) have been visualized bound to individual actin filaments with cryo-EM, providing insights into the overall structure of plastins and the actin-binding poses of single ABDs. A pioneering early electron microscopy study of negatively stained 2D paracrystalline F-actin arrays cross-linked by T-plastin provided a plausible model for the organization of a parallel bundle based on structural data available at the time (20) while leaving the detailed bundle formation mechanism undetermined.

Here, we present a single-particle cryo-EM workflow for visualizing the structure of cytoskeletal cross-linkers actively bridging filaments, enabled by the development of a machine-learning procedure to detect and presort candidate pairs of filaments with feasible three-dimensional (3D) cross-linking geometry. We employ this pipeline, along with supporting structural, biochemical, and cell biological studies, to uncover a mechanism enabling full-length T-plastin to sequentially engage two actin filaments, allowing it to form parallel and antiparallel bundles with nearly equivalent frequency.

## T-Plastin Initially Engages F-Actin through ABD2.

While to our knowledge no crystal structures of human plastins have been solved, T-plastin’s significant homology to previously crystallized *S. pombe* and *A. thaliana* fimbrins (49) (41.4% and 43.6% protein sequence identity, respectively) facilitated the calculation of a reliable homology model of its two tandem ABDs (Fig. 1*B*; see *Materials and Methods*), which we hereafter refer to as the prebound structure (i.e., yet to bind F-actin). This homology model is highly similar to the AlphaFold2 model (52) (AF-P13797; *SI Appendix*, Fig. S1), supporting the accuracy of homology modeling when a strong template is available. As reported in the crystal structures (49), prebound T-plastin adopts a closed horseshoe conformation with the N-terminal domain Calponin-Homology 1 (CH1) in close contact with the C-terminal CH4 (Fig. 1*B*). The two ABDs adopt a quasi-antiparallel orientation, connected by the 20-residue inter-ABD CH2–3 linker (Fig. 1 *A* and *B*, light green). Notably, the two intra-ABD linkers—the CH1–2 linker in ABD1 and the CH3–4 linker in ABD2—are very different in length (Fig. 1*A*, 28 residues and seven residues, respectively).

We next pursued structural studies of full-length human T-plastin bound to F-actin with cryo-EM in both the absence and presence of Ca^2+^ (*SI Appendix*, Fig. S2 and Table S1; *Materials and Methods*). Inspection of micrographs confirmed that although actin filaments are bundled under both conditions, bundling is suppressed by Ca^2+^ (*SI Appendix*, Fig. S2). Because the conventional iterative helical real space reconstruction (IHRSR) approach (53) can only be applied to single filaments, we initially focused on the +Ca^2+^ dataset to visualize the T-plastin–single F-actin interface. We obtained a 3D reconstruction using IHRSR as implemented in RELION 3.0 (54) (*SI Appendix*, Fig. S2 and Fig. 1*C*) at a global resolution of 2.6 Å. Local resolutions ranged from 2.4 Å to 3.9 Å, radially decaying from the core of the filament (*SI Appendix*, Fig. S3*A*), facilitating direct atomic model building and refinement for the complete sequence of Mg–adenosine phosphate α-actin and T-plastin residues 388 to 630 (Fig. 1*D* and *Materials and Methods*). Despite using the full-length protein, density for only two CHDs was observed (Fig. 1 *C* and *D*). The map resolution allowed us to unambiguously identify them as CH3 (olive) and CH4 (dark green), constituting ABD2 and a short segment of the CH2–3 linker (light green) at the N terminus of CH3 (Fig. 1*D*). The essentially uniform ABD2 decoration in the dataset indicates that when T-plastin engages F-actin, ABD2 must bind first. The robust actin binding by ABD2 furthermore indicates that Ca^2+^ suppresses T-plastin–mediated actin bundling by inhibiting the subsequent actin binding of ABD1 after ABD2 has engaged, rather than downregulating initial F-actin binding. This furthermore provides a structural rationale for previous biochemical studies demonstrating that Ca^2+^ does not inhibit plastin binding to individual actin filaments (47).

## Actin Binding Triggers Rearrangements within ABD2, Facilitating CH2–CH3 Inter-ABD Linker Docking.

Like many other ABPs (55), T-plastin’s CH3 domain of ABD2 engages a major site spanning the longitudinal interface of two adjacent actin protomers (Fig. 1*E*), which we term actin I and actin II (numbered from the plus/“barbed” end of the filament), while CH4 does not directly engage actin. CHDs are composed of four major α-helices (56), referred to here as H1 to H4 (Fig. 1*E*) from the N terminus to the C terminus, connected by loops and short, irregular helices. Helices H1, H3, and H4 constitute the actin-binding surface (ABS) of CH3, mediated by an extensive hydrophobic interface between H3, actin II, and actin I’s D-loop, as well as salt bridges between H1/H4 and actin I (Fig. 1*E*). Comparison of the actin conformation observed in a similar-resolution structure of F-actin in isolation (“bare actin”; PDB 7r8v) (57) versus when bound to T-plastin revealed minimal rearrangements throughout the actin structure (*SI Appendix*, Fig. S3*B*). Compared to the prebound T-plastin structure, CH3 maintains its overall conformation, featuring only slight remodeling in a loop (Fig. 1*F*), while its four major helices maintain their respective positions. However, CH4 undergoes an ∼30° swing around the CH3–4 linker to avoid a steric clash with actin (Fig. 1*F*), licensing a major rearrangement of the short stretch of the CH2–3 linker resolved in the cryo-EM map: a 180° flip away from the actin filament (Fig. 1*F*). Because this linker connects ABD2 to ABD1, actin engagement by ABD2 is anticipated to reposition ABD1.

Both CH3 and CH4 undergo minimal internal structural rearrangements when analyzed individually (*SI Appendix*, Fig. S3*C*), suggesting that interdomain movements through flexible linkers, rather than intradomain rearrangements of α-helices, predominate upon T-plastin’s actin binding. The three human plastin isoforms share highly similar sequences (*SI Appendix*, Fig. S4*A*) with at least 74% identity; consistently, our actin-bound full-length T-plastin structure is highly similar to the recently reported actin-bound structure of the truncated L-plastin ABD2 (PDB 6vec; *SI Appendix*, Fig. S4*B*) (27). Among all actin-bound tandem CHD protein structures (*SI Appendix*, Fig. S5*A*), only plastin ABD2’s second CHD could be resolved by cryo-EM (*SI Appendix*, Fig. S5 *B*–*D*), while the second CHDs of the utrophin, spectrin, and filamin A ABDs were not observed. This is likely because plastin ABD2 has the shortest intra-ABD linker between CHDs (*SI Appendix*, Fig. S5*A*), reducing the conformational flexibility of its actin-bound state (58). With the exception of plastin’s ABD2, tandem CHDs engage an additional minor actin-binding site through a short N-terminal extension that folds along F-actin (*SI Appendix*, Fig. S5 *B*–*D*). Plastin ABD2’s unique actin binding mode likely derives from its relative position in the protein sequence: Unlike other ABDs, plastin’s ABD2 is on the protein’s C-terminal end (Fig. 1*A*). Its preceding N-terminal element, the CH2–3 linker, engages the ABD2 itself upon actin binding and prevents it from forming an extended N-terminal actin-binding tail.

## A Frustrated, Prebundling State Sterically Necessitates Partial Disorder in ABD1.

Despite modest sequence identity and the length difference between their CH1–2 and CH3–4 intra-ABD linkers (*SI Appendix*, Fig. S5*A*), prebound ABD1 and ABD2 share an almost identical structure in their CHDs (Fig. 1*G*; rmsd 1.19 Å), indicating that when binding to F-actin, both ABDs are likely to employ a highly similar ABS (Fig. 1*G*), here annotated as ABS1 and ABS2 (Fig. 1*H*). However, the positioning of ABD1 in the prebound conformation is clearly incompatible with actin bundling, as ABS1 is sterically occluded within the core (Fig. 1*H*). This suggests a stepwise bundling mechanism in which initial actin binding by ABD2 triggers a conformational change that relieves auto-inhibition of ABD1, exposing ABS1 to bind a second actin filament.

**Fig. 2.**
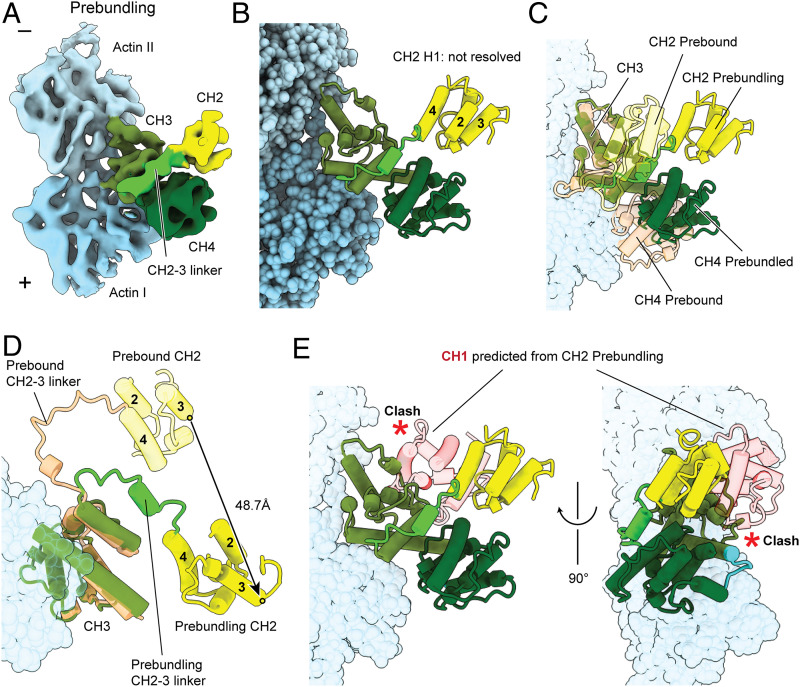
Identification of a prebundling conformation adopted by ABD1’s CH2. 3D classification of cryo-EM data collected in the absence of Ca^2+^ reveals a subpopulation featuring a partially ordered CH2 domain from ABD1. (*A*) Segmented region of the prebundling T-plastin–F-actin cryo-EM density map (6.9 Å resolution) in the absence of Ca^2+^ recovered by focused classification. (*B*) Flexible-fitting pseudoatomic model of the prebundling state. Actin subunits are displayed in varying shades of blue. CH2: yellow; CH2–3 linker: lime; CH3: olive; CH4: dark green. (*C*) Superimposed prebound and prebundling structures of T-plastin. (*D*) Same as (*C*), showing only CH2 H2–H4 and CH3. Circles represent the positions of D332’s Cα, whose displacement is displayed. (*E*) Superposition of the prebound ABD1 CH2 (not shown) on the prebundling CH2. The predicted position of CH1 results in a steric clash with ABD2, highlighted by red asterisks.

To test this hypothesis, we utilized the –Ca^2+^ dataset, where bundling is not suppressed, which we initially analyzed using IHRSR. Because widespread bundling reduced the number of filament segments per micrograph analyzable by IHRSR, we obtained a map at an overall lower (3.4 Å) resolution (*SI Appendix*, Fig. S2 and Table S1). Nevertheless, the atomic model derived from this map is indistinguishable from the +Ca^2+^ model, confirming that Ca^2+^ does not affect initial actin binding by T-plastin through ABD2 (*SI Appendix*, Fig. S3*A*). However, during 3D classification of the –Ca^2+^ dataset we noticed a weak signal distal from ABD2 in one class, suggestive of a partially ordered additional domain (*SI Appendix*, Fig. S2*A*, yellow circles), which was not present in any of the +Ca^2+^ classes at the same stage of processing. We therefore employed symmetry expansion to analyze each T-plastin binding site independently and extensive focused classification (*SI Appendix*, Fig. S6 *A* and *B*, yellow circle, and Table S2; *Materials and Methods*) to obtain a 6.9 Å reconstruction (Fig. 2*A* and *SI Appendix*, Fig. S6 *C* and *D*) of what we term a prebundling state consisting of the entire CH2–3 linker and the final three helices (H2–H4) of CH2 within ABD1. This map features clear helical density in CH2 (Fig. 2*A*), facilitating the use of docking and molecular dynamics flexible fitting (MDFF) to build a corresponding pseudoatomic model (*Materials and Methods*; Fig. 2*B* and *SI Appendix*, Table S2).

Aligning the prebound and prebundling states revealed a major conformational change within CH2 and the CH2–3 linker upon actin engagement (Fig. 2 *C* and *D*): the CH2-3 linker’s ∼120° swing results in a rotation and a 48.7 Å translation of CH2 (Fig. 2*D*). We next examined whether this conformational transition could directly prime ABD1 for engaging a second actin filament by repositioning it as a rigid body with ABS1 available for binding. Instead, the CH1 position predicted by aligning ABD1 from the prebound state with the portion of CH2 resolved in the prebundling state (Fig. 2*E*) produces a major steric clash with ABD2, a result that is incompatible with a priming model. Along with our observation that H1 of CH2 is not resolved, consistent with partial CH2 unfolding, this finding suggests that the prebundling reconstruction likely represents a frustrated, metastable state where a partially disordered ABD1 can sample a large conformational space to search for a second actin filament.

## Parallel and Antiparallel Bridges Feature Divergent ABD Orientations that Satisfy Linker Constraints.

Bundles of cytoskeletal filaments are not stoichiometrically defined protein complexes, and their geometry is incompatible with structural analysis by current IHRSR methods (53). To overcome these challenges, we implemented a workflow specifically for the structural analysis of filament bundles (*SI Appendix*, Fig. S7). First, we developed a machine-learning–based approach for specifically detecting pairs of actin filaments whose geometry is compatible with bridging by a cross-linking protein (*SI Appendix*, Figs. S6–S9 and *Materials and Methods*), the minimal unit of a cross-linked network. Briefly, we generated synthetic datasets of paired plastin-decorated actin filaments that uniformly sample a broad range of interfilament distances, relative orientations, and poses in projection views (*SI Appendix*, Fig. S8), which we used to train custom denoising auto-encoder and semantic segmentation neural networks (*SI Appendix*, Fig. S9). The semantic segmentation network allowed us to pick two-filament bundle particles from the –Ca^2+^ dataset, detecting both top views, where both filaments are clearly visible by eye, and side views, where the filaments visually overlap (*SI Appendix*, Fig. S10), supporting the feasibility of identifying all views required for 3D reconstruction.

To benchmark this procedure, we compared its performance to other recently developed neural network–based particle pickers supporting filament processing (Topaz [59] and crYOLO [60]) on a curated subset of micrographs (*Materials and Methods*). Instead of synthetic data, Topaz and crYOLO are intended to be trained on representative manually picked particles of the molecular species of interest. Indeed, we found that when trained on preselected top and side views of plastin-decorated two-filament bundles, crYOLO was able to successfully generate picks along the center of the bundle and was mostly successful in distinguishing two-filament bundles from single filaments in micrographs (*SI Appendix*, Fig. S11). When trained on the same manually picked particles, Topaz was less successful in providing well-centered picks and in distinguishing single filaments from two-filament bundles. Similarly, the template-based cross-correlation approach implemented in RELION was able to pick within bundled regions but failed to distinguish single filaments from two-filament bundles, and within two-filament bundles its picks were centered on individual filaments instead of bundles. Altogether, besides our bundle picker, crYOLO provided the best qualitative picks with the most quantitative similarity to our particle picker.

Thus, in principle we believe it would also be possible to use these programs to analyze cross-linkers, with crYOLO showing the most promise. However, as noted above, this would require initial manual picking of representative side views. This may be feasible in favorable cases, such as the reported structural analysis of cross-linker–independent, laterally associated doublets of the bacterial cytoskeletal filament ParM (61), where higher-order bundles were not observed and manual picking was employed successfully. However, it is likely to be challenging in practice for cases such as plastin, where two-filament bundles are frequently embedded in higher-order assemblies (*SI Appendix*, Fig. S10*A*). Our approach avoids the requirement for manually picking a training set; however, it does require prior structural information for the generation of synthetic training data, which may not always be available. Future structural studies of cross-linkers are likely to be enabled by a case-appropriate picking strategy, including that presented here, based on these considerations.

After picking and reference-free 2D classification, class averages were analyzed by projection-matching versus the plastin-decorated single filament map (*SI Appendix*, Fig. S12). This revealed both parallel and antiparallel actin bundles, thereby directly confirming that T-plastin cross-links actin filaments bidirectionally. Low-resolution initial models were generated by positioning two copies of the single-filament map in 3D via joint projection-matching against 2D class averages representing nearly orthogonal views. After 3D classification, selected parallel and antiparallel bundle particles were subsequently processed independently (*SI Appendix*, Fig. S13 *A*–*C*). These particles were subjected to extensive asymmetric 3D classification, uniform refinement, and finally multibody refinement in RELION 3.1 (*SI Appendix*, Fig. S13 *B* and *C* and Table S2 and Movies S1 and S2) to obtain final reconstructions of both parallel and antiparallel bundles featuring a single T-plastin bridge (*SI Appendix*, Fig. S13 *C* and *D*).

**Fig. 3.**
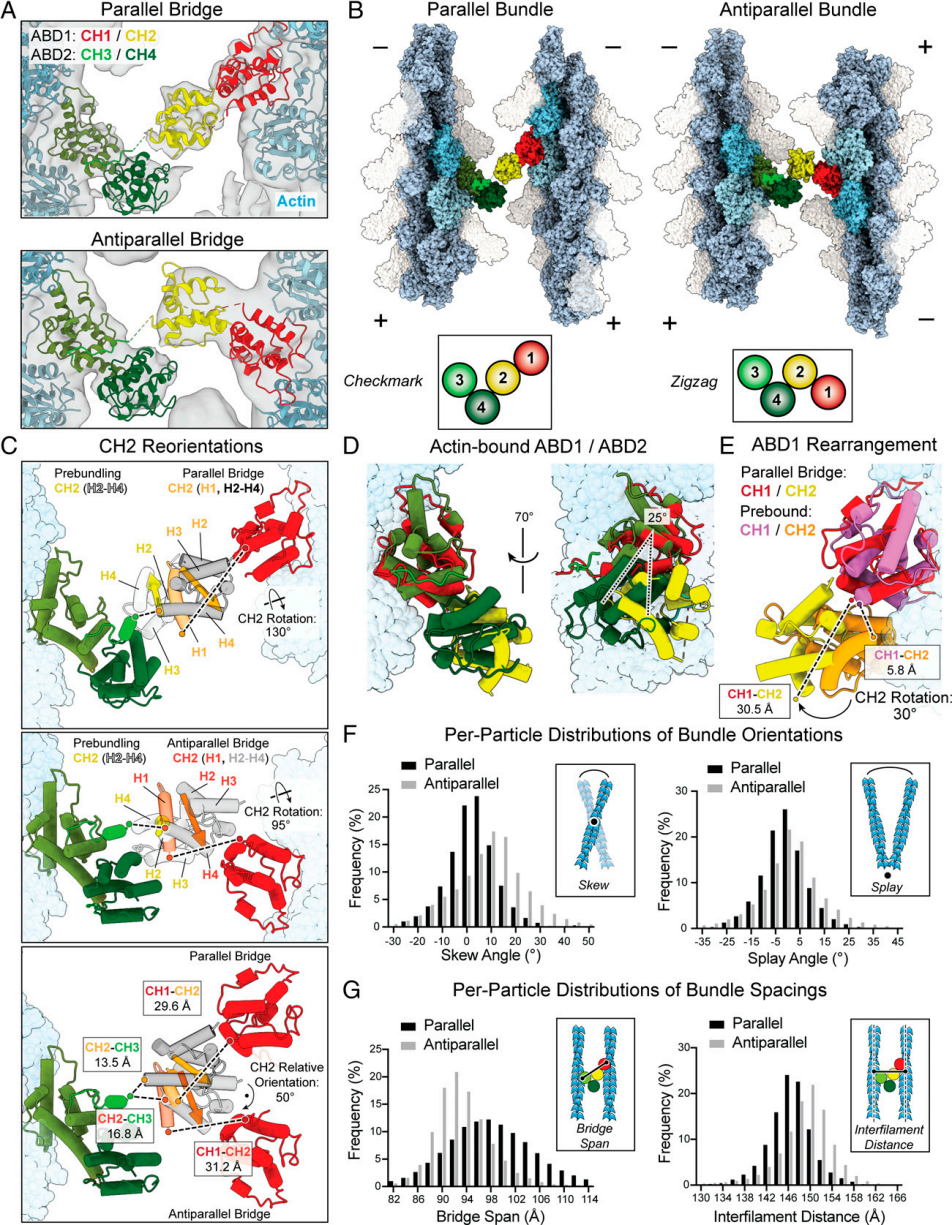
T-plastin flexibly bridges filaments in two conformations that satisfy linker constraints. Direct cryo-EM visualization of T-plastin molecules bridging actin filaments reveals parallel and antiparallel configurations at intermediate resolution. (*A*) Multibody refinement cryo-EM density maps of parallel (*Top*) and antiparallel (*Bottom*) two-filament bundles, with corresponding docking models of bridging T-plastin molecules colored as indicated. (*B*) Zoomed-out view of parallel and antiparallel bundle pseudoatomic models, highlighting their distinct T-plastin bridge conformations. (*C*) Superpositions of the prebundling state with the parallel bridge (*Top*) and antiparallel bridge (*Middle*), as well as the parallel and antiparallel bridges (*Bottom*). CH2 H1 is highlighted, and CH2 reorientations as well as inter-CH linker lengths are annotated. Vectors from I309 to P363 indicate the overall orientation of CH2. (*D*) Superposition of the ABD1 and the ABD2 actin-binding interfaces in the parallel bundle, aligned on actin, highlighting their distinct poses relative to the filament. (*E*) Superposition of ABD1 from the prebound and parallel bundle models. The extension of the CH1–2 linker (240–269) and the reorientation of CH2 relative to CH1 upon actin binding are displayed. (*F*) Per-particle distributions of indicated bundle orientation angles measured through docking analysis of multibody refinement results. Parallel, *n* = 41,701; Antiparallel, *n* = 28,759. (*G*) Same as (*F*), but distributions of indicated bundle spacing parameters.

To interpret these maps, we compared them with the postbound ABD2 map low-pass filtered to 9 Å resolution (*SI Appendix*, Fig. S14). By inspection, the ABD densities from multibody refinement of the parallel bundle were clearly distinct, with only body 2 appearing highly similar to the postbound ABD2 reconstruction. Consistently, docking analysis with the postbound ABD2 model produced a higher cross-correlation score for body 2. Although somewhat more subtle for the antiparallel bundle, visual inspection and docking analysis once again identified a single body as ABD2 (body 1). These assignments facilitated building models of the ABD1–ABD2 core in both bridging conformations (Fig. 3*A*). While quantitative Fourier Shell Correlation (FSC) assessment supports overall subnanometer resolution of these reconstructions (*SI Appendix*, Table S2), in both cases the bridging plastin density does not uniformly contain well-defined secondary structure features (Fig. 3*A*). This might be due to residual plastin flexibility relative to the bound actin filaments, as well as moderate resolution anisotropy (*SI Appendix*, Fig. S13*D*) from a mild preferred orientation enriching top views (*SI Appendix*, Fig. S13*A*). Additionally, the antiparallel conformation features rotational pseudosymmetry between the two ABDs, increasing the likelihood of misaligned particles. The maps could nevertheless be modeled using a combined approach of rigid-body docking and MDFF (*Materials and Methods*; *SI Appendix*, Table S2), which has successfully been employed in similar cases (62). These models are suitable for interpreting gross structural properties (e.g., folding status) and orientations of individual CH domains.

In both bundle configurations, ABD1 and ABD2 bind their respective filaments in a similar fashion, with CH1/CH3 both oriented toward the filament minus ends (Fig. 3*B*). As a result, when bridging a parallel bundle, T-plastin’s actin-binding core adopts a configuration resembling a checkmark (Fig. 3*B*, *Left*), whereas the antiparallel bridge resembles a zigzag (Fig. 3*B*, *Right*). Superimposing ABD1 and ABD2 from the parallel bridge shows that both ABDs bind a nearly identical site on F-actin (Fig. 3*D*), confirming our ABS prediction based on the high degree of structural homology between CH1 and CH3 (Fig. 1*G*). However, the two ABDs protrude at angles that differ by ∼25° relative to the actin filament’s central axis (Fig. 3*D*), a finding validated by a contemporary cryo-EM study of the isolated ABD1 bound to F-actin (51), resulting in asymmetric actin bundling by T-plastin.

We next compared the prebundling state to both bundled states to examine how two nearly orthogonal cross-linking geometries can be achieved (Fig. 3*C* and Movies S3–S4). In both cases, we observed major rotational rearrangements around the CH2–CH3 inter-ABD linker, repositioning CH2 such that its H1 helix can refold, with the helix in a similar position despite the rest of the CHD adopting diverging orientations (Fig. 3*C*). In both cases, this produces a highly extended state of the long CH1–CH2 intra-ABD linker, which was not resolved in our reconstructions. Indeed, comparing the prebound and parallel bridge postbound ABD1 conformations shows a striking rearrangement, with an ∼30° relative rotation of CH1 versus CH2 (Fig. 3*E*). While this conformation enables both actin engagement by CH1 and H1 redocking on CH2, it also requires a substantial increase in the distance between the CH1–CH2 linker’s N terminus and C terminus, from 5.8 Å to 30.5 Å. Although this distance can readily be spanned by the 28 residue linker, we speculate that this will disrupt its compact, partially folded conformation observed in the prebound state (Fig. 1*B*), consistent with it becoming flexible and unresolved in our bundle reconstructions. We thus speculate that the checkmark and zigzag conformations are the two bridging configurations that satisfy the spatial constraints imposed by the CH2–CH3 and CH1–CH2 linkers while also enabling CH2 H1 to refold, both of which can be accessed with approximately equal probability due to the structural flexibility of ABD1 in the prebundling state. Consistent with this model, we observed that the end-to-end distances spanned by the unresolved segments of the CH2–CH3 linker (parallel: 13.5 Å; antiparallel: 16.8 Å) and CH1–CH2 linker (parallel: 29.6 Å; antiparallel: 31.2 Å) are highly similar between the two bridge conformations, despite a 50° difference in CH2’s orientation (Fig. 3*C*, *Bottom*).

## T-Plastin Forms Flexible Cross-Links within Polarity-Specific Geometric Constraints.

As the CH1–CH2 linker and a portion of the CH2–CH3 linker were not resolved in either bundle reconstruction and we observed substantial resolution enhancements in both reconstructions after multibody refinement (*SI Appendix*, Fig. S13*D*), we hypothesized that both configurations would feature conformational variability. To explicitly map the conformational landscape of the T-plastin bridges in our data, we implemented a high-throughput procedure to analyze the distribution of bundle geometries present. Briefly, we utilized RELION’s capacity to generate a 3D reconstruction featuring the optimal positioning of each filament detected in every bundle particle during multibody refinement, followed by automated rigid-body docking of decorated filament atomic models whose relative positioning we subsequently analyzed (*Materials and Methods*). We assigned a common 3D frame of reference and defined two angles between the filaments to describe a bundle: skew, the out-of-plane tilt, and splay, the in-plane rotation (Fig. 3*F*). We also defined two spacing parameters: bridge span, the distance across a bridging T-plastin molecule, and interfilament distance, the shortest distance between the central axis of each filament (Fig. 3*G*).

Both configurations feature broad, unimodal distributions of all four parameters, with no apparent correlation between skew and splay (*SI Appendix*, Fig. S15*A*), consistent with T-plastin acting as a freely flexing joint within its allowable conformation space, thereby accommodating diverse bundling geometries in the presence of mechanical perturbations. However, the conformational landscape of each configuration is distinct (Fig. 3 *F* and *G*). Both the splay (mean = −1.0°; SD = 9.1°) and skew (mean = 2.4°; SD = 9.7°) distributions of the parallel bundle are centered around ∼0°, suggesting that the checkmark configuration preferentially engages collinear filaments. In contrast, while the splay angle distribution of the antiparallel bundle is also approximately centered around 0° (mean = 1.1°; SD = 12.4°), the center of its skew angle distribution is substantially displaced (mean = 9.0°; SD = 14.2°). These data suggest that the zigzag conformation instead preferentially bridges filaments that deviate from collinearity. This result offers an explanation for why highly collinear actin bundles cross-linked by plastin (e.g., stereocilia and microvilli) often exclusively feature parallel filaments (10), where many bridging plastin molecules in the checkmark conformation could accumulate along neighboring filaments to reinforce this network geometry. However, mesh-like networks (e.g., the cell cortex and lamellipodia) frequently feature mixtures of parallel and antiparallel filaments (63), where our data suggest that the incorporation of zigzag antiparallel bridges would disfavor collinearity. Thus, T-plastin may preferentially adopt the checkmark or zigzag conformation in specific subcellular contexts. Despite these distinct angular preferences, both conformations feature similar bridge spans (parallel mean: 98.2 Å; antiparallel mean: 93.1 Å) and interfilament distances (parallel mean: 146.4 Å; antiparallel mean: 148.9 Å), which are constrained by the dimensions of the T-plastin molecule, as predicted from previous structural analyses (20, 49). When the maximal width of F-actin is considered (∼70 Å), the remaining space between filaments also closely matches the width of a single filament. Our data thus suggest that plastin is optimal for cross-linking dense networks, where gaps of this span should predominate (63, 64).

## Linker Rearrangements Underlie T-Plastin’s Sequential Actin Bundling Mechanism.

**Fig. 4.**
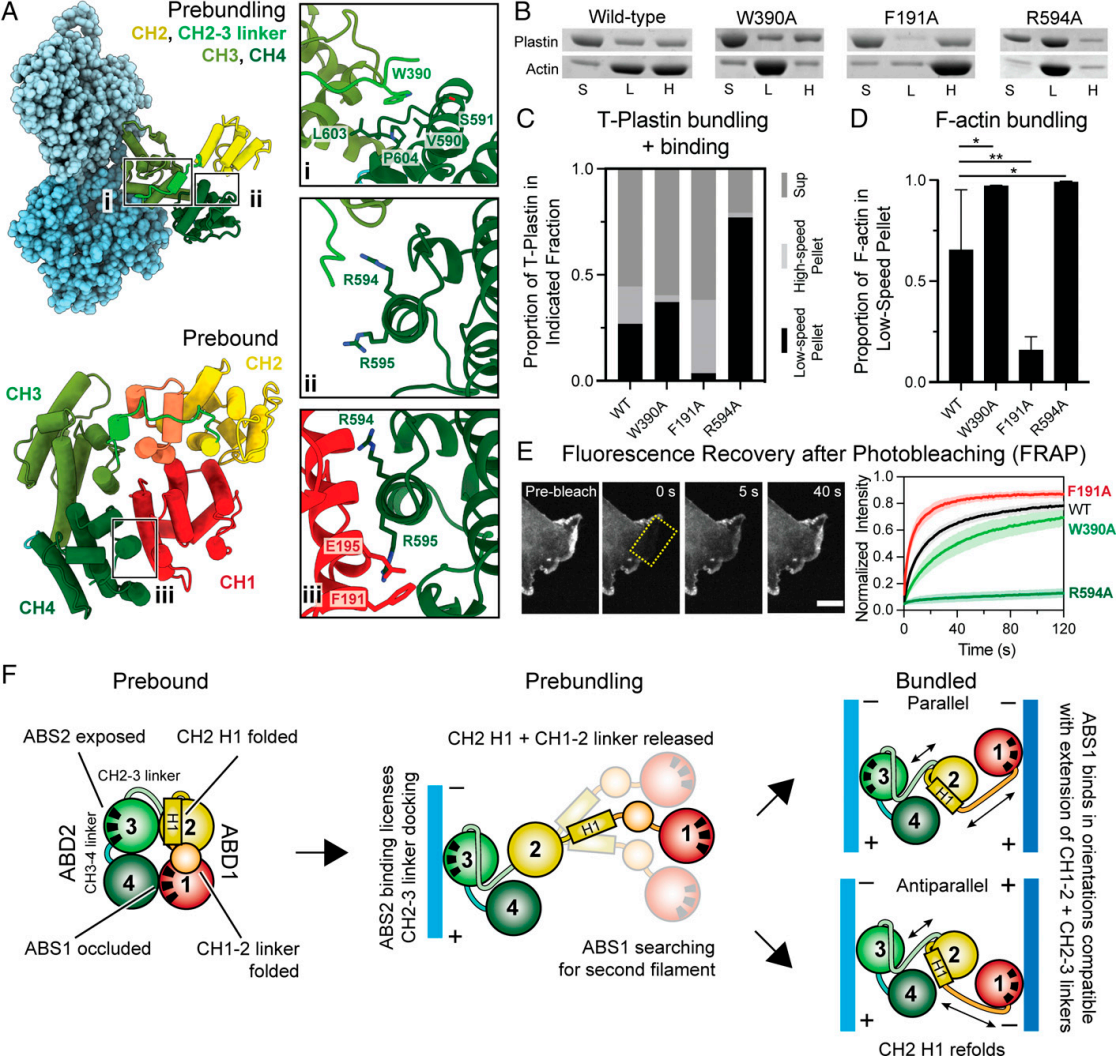
T-plastin employs a sequential mechanism to bidirectionally cross-link actin filaments. The behavior of T-plastin point mutants in actin binding and bundling assays is consistent with a sequential cross-linking mechanism. (*A*) T-plastin residues targeted for site-directed mutagenesis in the CH2–3 linker (i), at the prebound CH1–CH4 binding interface (ii, iii), and in ABS1 (iii). (*B*) Representative denaturing polyacrylamide gel electrophoresis analysis of sequential low-speed/high-speed F-actin cosedimentation assays. S, supernatant; L, low-speed pellet; H, high-speed pellet. (*C*) Quantification of (*B*): proportions of indicated T-plastin constructs in each fraction. 4 ≤ *n* ≤ 7. See *SI Appendix*, Fig. S16 for statistical analysis. (*D*) Quantification of (*B*): proportion of F-actin in low-speed pellet (indicative of bundling) (Error bars, SD). Wild type (WT): *n* = 7; mutants: *n* = 4. WT/W390A: *P* = 0.04; WT/F191A: *P* = 0.006; WT/R594A: *P* = 0.03. *P < 0.05, **P < 0.01, two-tailed *t* test. (*E*) FRAP assays in live HUVEC cells of the indicated GFP tagged T-plastin constructs (Scale bar, 10 μm). (*F*) Conceptual mechanistic model.

Our structural data collectively suggest that T-plastin employs a sequential bundling mechanism, where initial actin engagement by ABD2 triggers rearrangements in the CH2–CH3 inter-ABD linker, which docks on ABD2 to release ABD1 from an occluded, auto-inhibited state, enabling it to bind a second filament. To test this model, we designed a series of structure-guided point mutants and examined their actin binding and bundling activities. In our high-resolution actin-bound structure, CH2–CH3 linker residue W390 engages a series of hydrophobic residues along CH3 H1 (Fig. 4*A*), suggesting a role in mediating the docking of the CH2–CH3 linker after ABD2 initially engages a filament. To test this hypothesis, we generated a W390A mutant to perturb this interface and examined its actin-binding and bundling activities via a sequential low-speed, high-speed actin cosedimentation assay in which the plastin-dependent formation of multifilament bundles, which sediment at a lower relative centrifugal force (RCF), is distinguishable from plastin binding to individual filaments (65) (*Materials and Methods*; Fig. 4 *B*–*D*). In our assay conditions (5 μM actin, 2 μM plastin), wild-type T-plastin was ∼50% bound to actin (Fig. 4 *B* and *C* and *SI Appendix*, Fig. S16), and actin along with engaged T-plastin was nearly evenly divided between the low-speed and high-speed pellets (Fig. 4 *B* and *D*), consistent with a mixture of bundles and individual filaments. Although the overall bound fraction of the W390A mutant was indistinguishable from wild type (Fig. 4 *B* and *C* and *SI Appendix*, Fig. S16), F-actin was significantly shifted to being nearly 100% in the low-speed pellet fraction (Fig. 4 *B* and *D*). While W390A increasing bundling is somewhat counterintuitive, it is possible that once ABD1 is released, tight CH2–CH3 linker docking restricts the conformational flexibility of bridging plastin molecules. Interpreted in this framework, W390A could enhance bundling by licensing a broader distribution of bridge conformations and corresponding bundle orientations than the wild type. Regardless, this inter-ABD linker mutation having a specific effect on bundling is broadly consistent with the sequential mechanism.

In the prebound conformation, CH4 forms an extensive binding interface with CH1’s ABS (Fig. 4*A*), which we hypothesize stabilizes the auto-inhibition of ABD1. The sequential mechanism predicts that disrupting this interface by mutating CH4 residues should enhance T-plastin’s binding and bundling activities by facilitating the release of ABD1, while mutating CH1 residues should conversely suppress bundling by reducing binding to a second filament through ABD1. Consistent with this hypothesis, CH4 mutants R594A (Fig. 4 *B*–*D*) and R594A R595A (*SI Appendix*, Fig. S17) both bind and bundle actin significantly more than the wild type, with both mutants and actin found almost exclusively in the low-speed pellet, while CH1 ABS mutant F191A diminishes bundling yet nevertheless strongly binds individual filaments (Fig. 4 *B–D*), with a total actin-bound fraction indistinguishable from wild type (*SI Appendix*, Fig. S16). However, the R595A mutation alone has minimal effect (*SI Appendix*, Fig. S17), indicating that alanine substitutions in this region do not nonspecifically disrupt T-plastin’s actin binding and bundling activities.

To assess the significance of the sequential bundling mechanism in cells, green fluorescent protein (GFP)-tagged wild-type and mutant T-plastins were expressed in human umbilical vein endothelial cells (HUVECs) to image their subcellular localizations and dynamics (*SI Appendix*, Fig. S17*A*). While wild-type T-plastin and most mutants examined primarily colocalized with the ARP2/3 complex at lamellipodia, suggesting a preference for branched networks at cellular protrusions as recently reported (8), the enhanced bundling mutants R594A and R594A R595A exhibited a striking shift, displaying substantial localization at stress fibers and focal adhesions, where collinear filaments predominate. A similar relocalization phenotype was reported for the phosphomimetic S406E L-plastin mutant, which is also anticipated to disrupt inter-ABD association (51). Collectively, these data suggest that plastin auto-inhibition and the sequential bundling mechanism are critical for actin network selection, thereby governing subcellular localization. We next pursued fluorescence recovery after photobleaching (FRAP) assays in cells where F-actin assembly and disassembly were pharmacologically arrested, facilitating the specific probing of plastin binding dynamics (*Materials and Methods*; Fig. 4*E* and *SI Appendix*, Fig. S17). In these studies, we interpreted rapid recovery to broadly correspond with low bundling activity, indicating that T-plastin is readily displaced from actin networks in vivo while slow recovery is conversely indicative of strong bundling activity (8). Consistent with our in vitro binding assays, W390A showed significantly slower recovery than the wild type (Fig. 4*E*), with a half-time (t_1/2_) of 34.6 versus 17.7 s (*SI Appendix*, Table S3), indicative of enhanced bundling. Conversely, F191A displayed more rapid recovery (Fig. 4*E*; t_1/2_ 8.8 s; *SI Appendix*, Table S3), indicative of weaker bundling. The R594A (Fig. 4*E*; t_1/2_ 212.9 s, ∼75% immobile; *SI Appendix*, Table S3) and R594A R595A mutants (*SI Appendix*, Fig. S17) both showed minimal recovery, indicative of an extremely stable engagement of actin networks, while the R595A mutant alone once again had minor effects (*SI Appendix*, Fig. S17). In summary, the sequential mechanism broadly predicted the impacts of T-plastin mutations both up-regulating and down-regulating bundling in vitro and in cells.

To assess whether the model could also rationalize osteoporosis-linked T-plastin mutations reported in human patients, we mapped these mutations onto our prebundling and postbundling structures (*SI Appendix*, Fig. S15*B*). Other than two mutations in CH3, N446S and L478P, that likely impact the ABD2–actin binding interface (27), all other mutations cluster at the center of the T-plastin bridge in the regions coordinating the structural transitions we describe here, including interfaces between CH4 and CH2, and the CH1–CH2 linker. This finding suggests that their pathophysiology could feasibly be linked to disruption of the conformational transitions required for regulated bidirectional F-actin bridging.

## Discussion

Here, we introduce a method for visualizing cytoskeletal filaments bridged by cross-linking proteins, which we have used to uncover a mechanism enabling the evolutionarily ancient family of plastin/fimbrin tandem CHD proteins to bidirectionally cross-link actin filaments. Our structural, biochemical, and cell biological data support a sequential actin bundling mechanism (Fig. 4*F*), in which structural transitions in the flexible linkers between T-plastin’s four CHDs facilitate conformational contortions that enable the protein to stably engage two nearly oppositely oriented filament geometries. We speculate that the formation of the metastable, single-filament–engaged prebundling state allows ABD1 to conduct a broad search for a second filament. This can resolve into two stable bridging conformations, the parallel checkmark and antiparallel zigzag, likely due to a complex balance of forces exerted through linker dynamics, CH2 H1 folding, and ABD1 actin binding. While having both ABDs engage actin will stabilize a bridge, CH2 H1 refolding requires a CH2 orientation that substantially extends the long CH1–CH2 linker, which is likely unfavorable versus its folded configuration in the prebound state (Fig. 4*F*). Thus, while our multibody analysis clearly supports substantial flexibility in both plastin bridge conformations (Fig. 3 *F* and *G*), we speculate that the CH1–CH2 linker acts as an internal spring-like element that restricts their conformational landscapes.

While this work was under review, a contemporary study by Schwebach et al. reported extensive biochemical characterization of the interplay between plastin’s ABD1 and ABD2 (51). Isolated ABD2 was found to possess intrinsic nanomolar affinity for F-actin, which could be allosterically inhibited in trans by ABD1. Isolated ABD1, on the other hand, displayed micromolar affinity for F-actin. While these observations also led Schwebach et al. to formulate a sequential binding mechanism, they proposed that ABD1 binds F-actin first based on prior indirect biochemical evidence (27). We believe that these data are also highly compatible with the mechanism we propose here (ABD2 binding first) based on direct structural observations. In this framework, the stable and long-lived ABD2–F-actin interaction licensed by ABD1 release would facilitate ABD1’s search for a second filament, producing efficient bundling. Once in a bridging conformation, the high local concentration of F-actin binding sites for ABD1 would facilitate the maintenance of stable cross-linking, even if ABD1 were to briefly dissociate and then re-engage. This is particularly relevant in the case of parallel filaments, where the filament geometry is compatible with multiple plastin molecules engaging along a local bundle region. Conversely, if ABD1 were to bind first, then its weak interaction and fast dissociation would require ABD2 to immediately engage a second filament, without the opportunity for a prolonged search.

Our finding that ABD2 is the first to bind F-actin also provides a structural rationale for the lack of Ca^2+^ regulation of plastin’s binding to single filaments, as it is distal from the RD. However, because the RD and bound Ca^2+^ ions were not resolved in our studies (likely due to flexibility), our structures do not directly inform upon the mechanism of bundling suppression by Ca^2+^. Nevertheless, the sequential mechanism presented here is fully compatible with a previously proposed model in which the RD engages and down-regulates the actin binding of ABD1 in the presence of Ca^2+^ to suppress bundling (48). Previous biochemical characterization of purified osteoporosis disease mutants A368D (located in CH2) and E249_A250insI-L/A253_L254insN (located in the CH1–2 linker) demonstrated that these lesions specifically disrupt Ca^2+^ regulation without impairing binding or bundling in the absence of Ca^2+^ (27). These mutations are not located in ABS1 but instead map to ABD1 regions, which remodel as plastin adopts the bridging conformations we describe here (Fig. 3*E* and *SI Appendix*, Fig. S15*B*). We therefore speculate that the Ca^2+^-bound RD allosterically interferes with ABD1’s ability to stably assume the bridging conformations needed to engage a second filament without impairing actin binding through ABD2.

Our studies provide a structural framework for plastin’s versatility in stabilizing dense actin networks with multiple filament polarity organizations, likely an important feature for an ancestral cross-linker family required to broadly fulfill actin cross-linking functions in ancient eukaryotes. The diversification of cross-linkers featuring CHD actin-binding domains, most of which form well-defined homomers with stricter geometric requirements (e.g., α-actinins, spectrins, filamins) (56), thus likely tracked with the capacity of complex cells to build subcellular actin networks with specialized properties. Our analysis highlights how nanoscale structural transitions in a cross-linking protein can dramatically alter the mesoscale geometric properties of the actin network that it builds, and other cross-linkers may also populate complex structural landscapes that impact cytoskeletal self-organization.

## Materials and Methods

Cryo-EM specimens were prepared from mixtures of 0.6 μM F-actin and 20 μM T-plastin in the presence and absence of saturating Ca^2+^ using a published protocol (26) and then were imaged with Titan Krios cryo-transmission electron microscopes (ThermoFisher/FEI) operating at 300 kV using Gatan K2 Summit direct electron detectors. Both the +Ca^2+^ and –Ca^2+^ postbound states were reconstructed using a standard RELION IHRSR workflow (53, 54) as previously described (26), and the –Ca^2+^ prebundling state was subsequently recovered through symmetry expansion followed by extensive focused classification in RELION. Custom machine-learning software was used to pick two-filament bundles in the –Ca^2+^ dataset that were subsequently structurally analyzed using single-particle approaches and RELION multibody refinement. T-plastin mutants’ binding and bundling of F-actin were biochemically examined in vitro using cosedimentation assays as previously described (65), as well as in cells using fluorescence microscopy and FRAP assays. Detailed descriptions of experimental and computational methods are provided in *SI Appendix*, *Materials and Methods*.

## Supplementary Material

Supplementary File

Supplementary File

Supplementary File

Supplementary File

Supplementary File

## Data Availability

The atomic coordinates for T-plastin–F-actin complexes have been deposited in the Protein Data Bank with the following accession codes: 7R94 (66), high-resolution postbound model; 7SXA (67), prebundling model; 7SX8 (68), parallel bundle model; and 7SX9 (69), antiparallel bundle model. Cryo-EM density maps have been deposited in the Electron Microscopy Data Bank with the following accession codes: EMD-24323 (70), 2.6 Å high-resolution map (+Ca^2+^); EMD-25496, 3.4 Å high-resolution map (–Ca^2+^); EMD-25496 (71), prebundling map; EMD-25494 (72), parallel bundle map; and EMD-25495 (73), antiparallel bundle map. Custom software is available at https://github.com/alushinlab/plastin_bundles (74) as open source. All other data are included in the manuscript and/or *SI Appendix*.
