# Supplementary material for: Structural mechanism for bidirectional actin cross-linking by T-plastin
Source: Proc Natl Acad Sci U S A. 2022 Sep 6;119(37):e2205370119. doi: 10.1073/pnas.2205370119 (PMC9478642; doi:10.1073/pnas.2205370119)
Supplement: Supplementary File [file pnas.2205370119.sapp.pdf]

PNAS

Supplementary Information for
Structural mechanism for bidirectional actin cross-linking by T-plastin.

Lin Mei, Matthew J. Reynolds, Damien Garbett, Rui Gong, Tobias Meyer, Gregory M. Alushin

Gregory M. Alushin
Email: galushin@rockefeller.edu

This PDF file includes:

Supplementary Text
Figures S1 to S17
Tables S1 to S3
Legends for Movies S1 to S5
SI References

Other supplementary materials for this manuscript include the following:

Movies S1 to S4

Supplementary Information Text

SI Materials and Methods

Cloning, Expression, and purification of T-plastins

Sequences for the WT, full-length human T-plastin (1-630), as well as T-plastin mutants F191A, W390A, R594A, R595A, and R594 R595A were inserted into a pE-SUMO vector with an N-terminal His6-tag and a SUMO-tag. Sequence of the SUMO protease Ulp1 was inserted into a pET vector with an N-terminal His6-tag. All T-plastin constructs and the SUMO protease Ulp1 were expressed in Rosetta2(DE3) E. coli cells (Novagen) grown in LB media at 37°C to an optical density of 0.8-1.0 and induced with 0.7 mM IPTG. After induction, the cells were grown for 16 hours at 16°C, then cell pellets were collected and stored at -80°C until use. Cell pellets were resuspended in Lysis Buffer (50 mM Tris-Cl pH 8.0, 150 mM NaCl, 5% v/v glycerol, 2 mM β-mercaptoethanol, 20 mM imidazole) and lysed with an Avestin Emulsiflex C5 homogenizer, after which the lysate was clarified at 15,000 g for 30 minutes. Cleared lysate was incubated with Ni-NTA resin (Qiagen) for 1 hour on a rotator at 4°C, after which the flow-through was discarded and the resin was washed with 5 bed volumes of lysis buffer. Proteins were subsequently eluted in Elution Buffer (50 mM Tris-Cl pH 8.0, 150 mM NaCl, 5% v/v glycerol, 2 mM β-mercaptoethanol, 300 mM imidazole). Purified His-tagged SUMO protease Ulp1 was then added at 0.05 mg / ml working concentration into the protein solutions of SUMO-tagged proteins, then the protein solution was dialyzed against Dialysis Buffer (20 mM Tris-Cl pH 8.0, 300 mM NaCl, 5% v/v glycerol, 2 mM β-mercaptoethanol) for 16 hours. The protein solution was then reapplied to Ni-NTA resin, and the flow-through was collected. Protein was then sequentially purified by a HiTrapQ HP anion exchange column (GE Healthcare) followed by size exclusion chromatography on a Superdex 200 Increase column (GE Healthcare) in Gel Filtration Buffer (20 mM Tris-Cl pH 8.0, 150 mM NaCl, 5% v/v glycerol, 2 mM β-mercaptoethanol) supplemented with 10% v/v glycerol, then snap-frozen in liquid Nitrogen and stored at -80°C.

Cryo-EM sample preparation and data collection

Filamentous actin (F-actin) was polymerized in G-Mg (2 mM Tris-Cl pH 8.0, 0.5 mM DTT, 0.2 M ATP, 0.01% NaN_3, 0.1 mM $MgCl_2$) and KMEI (50 mM KCl, 1 mM $MgCl_2$, 1 mM EGTA, 10 mM imidazole pH 7.0, 1mM DTT) buffers as described previously (*1*) from 5μM globular actin (G-actin) monomers at room temperature for 1 hr, then diluted to 0.6 μM in KMEI prior to use. Full-length human T-plastin was buffer exchanged into KMEI or Ca-KMEI (KMEI buffer + 2mM $CaCl_2$) by desalting column (GE PD SpinTrap G-25) and was maintained at a concentration of 20 μM. All KMEI buffers were supplemented with 1 mM DTT and 0.05% NP-40.

Immediately prior to sample preparation, CF-1.2/1.3-3Au 300-mesh gold C-flat holey carbon cryo-TEM grids (Protochips) were plasma cleaned with a Hydrogen / Oxygen mixture for 5 seconds in a Gatan Solarus. Actin solution (3 μl) was first applied to the grid in the humidified chamber of a Leica EM GP plunge freezer and incubated for 60 s at 25°C. T-plastin solution (3 μl) was then applied and incubated for 30 s. Solution (3 μl) was then removed and an additional 3 μl of T-plastin solution was applied. After an additional 30 s, the grid was back-blotted for 5 s, plunge-frozen in ethane slush, and stored in liquid Nitrogen until imaging.

Cryo-EM data for the T-plastin–actin (–Ca^{2+}) complex were recorded on a Titan Krios (ThermoFisher / FEI) at the Rockefeller University operated at 300 kV equipped with a Gatan K2 Summit camera. SerialEM (*2*) was used for automated data collection. Movies were collected at a nominal magnification of 29,000X in super-resolution mode resulting with a calibrated pixel size of 1.03 Å / pixel (super-resolution pixel size of 0.515 Å / pixel), over a defocus range of -1.5 to -3.5 μm. 40 frames were recorded over 10 s of exposure at a dose rate of 6 electrons per pixel per second (1.5 electrons per $Å^2$ per second) for a cumulative dose of 60 electrons per $Å^2$. The T-plastin–actin (+Ca^{2+}) dataset was recorded at the New York Structural Biology Center (NYSBC) on a Titan Krios operated at 300 kV also equipped with a Gatan K2 Summit camera. Movies were collected in counting mode resulting in a calibrated pixel size of 1.06 Å / pixel, over a defocus range of -1.5 to -3.5 μm. 50 frames were recorded over 10 s of exposure at a dose rate of 1.34 electrons per pixel per second (1.42 electrons per $Å^2$ per second) for a cumulative dose of 71.02 electrons per $Å^2$.

High-resolution cryo-EM image processing

Unless otherwise noted, all image processing was performed within the RELION-3.0 package (*3*), following a recently described procedure (*4*). Movie frames were aligned and summed with 2 x 2 binning for the $–Ca^{2+}$ dataset, and no binning for the $+Ca^{2+}$ dataset, using the MotionCor2 algorithm (*5*) implemented in RELION (*6*) with 5 × 5 patches. The contrast transfer function (CTF) was estimated from non-doseweighted summed images with CTFFIND4 (*7*). Bimodal angular searches with psi angle priors were used in all subsequent 2D and 3D alignment / classification procedures. Approximately 2,000 segments were initially manually picked, extracted, and subjected to 2D classification to generate templates for auto-picking. Helical auto-picking was then performed with a step-size of 3 asymmetric units corresponding to a 27 Å helical rise. Segments were extracted from dose-weighted (*8*) sum images in 512 x 512 pixel boxes without down-sampling, followed by a second round of 2D classification and auto-picking with featureful class averages. Auto-picked segments were then extracted and subjected to 2D classification using a 200 Å tube diameter and 300 Å mask diameter. Segments that contributed to featureful class averages were selected for 3D analysis.

All subsequent 3D analysis steps were primed with estimates of helical rise and twist of 27.0 Å and -167.0°, respectively, utilizing an initial reference low-pass filtered to 35 Å resolution, with the outer tube diameter set to 200 Å, inner tube diameter set to -1, and the mask diameter set to 300 Å. The first round of 3D classification into 3 classes was performed with bare actin filament reconstruction (EMBD-7115) as the initial reference. A second iteration was then performed with a class featuring clear ABP density from the first round as the initial reference. For the T-plastin–actin ($–Ca^{2+}$) dataset, this second round of 3D classification yielded two classes with helical parameters similar to the initial estimates and well-resolved 3D features, and one junk class with aberrant helical parameters and distorted features. For T-plastin–actin ($+Ca^{2+}$) dataset, all three classes were good, featureful 3D classes, indicating the high quality of this dataset. Segments contributing to the selected classes were then pooled for 3D auto-refinement.

The first round of auto-refinement was then performed using one good 3D class as the initial reference. All masks for subsequent post-processing steps were calculated with 0 pixel

extension and a 6 pixel soft edge from the corresponding converged reconstruction, low-pass filtered to 15 Å and thresholded to fully encompass the density. First-round post-processing was performed with a 50 % z length mask, followed by CTF refinement without beam-tilt estimation and Bayesian polishing (*6*). A second round of auto-refinement was then performed, followed by post-processing with a 30 % z length mask, then a second round of CTF refinement with beam-tilt estimation and Bayesian polishing. Final auto-refinement was then performed, once again employing a 30 % z length mask for post processing.

The final reconstructions converged with helical rise of 27.0 Å and twist of -166.8° for the T-plastin–F-actin ABD-F-actin complex (–Ca^{2+}), and a helical rise of 27.4 Å and twist of -166.8° for the T-plastin–F-actin ABD-F-actin complex (+Ca^{2+}), consistent with our finding that actin rearrangements evoked by T-plastin are minimal (*SI Appendix*, Fig. S3B). Global resolution estimates were 3.4 Å for the full-length T-plastin–F-actin complex (–Ca^{2+}), and 2.6 Å for the full-length T-plastin–F-actin complex (+Ca^{2+}), by the gold-standard Fourier shell correlation (FSC) 0.143 criterion. The B-factors estimated during post-processing were then used to generate sharpened, local-resolution filtered maps with RELION. Key statistics summarizing high-resolution cryo-EM image processing are reported in *SI Appendix*, Table S1.

T-plastin pre-bundling state cryo-EM image processing

The weak signal in Class I of the –Ca^{2+} condition (*SI Appendix*, Fig. S2) suggested that in a subset of the particle images, signal from an additional CH domain was consistently present in a defined region. To isolate and refine those particles, symmetry expansion and subsequent rounds of focused 3D classification were used to generate a set of 322,743 segment images (*SI Appendix*, Fig. S6A). Using the asymmetric unit and helical parameters from 3D auto-refinement, particles were symmetry expanded. These particles were then re-extracted with recentering of 2D shifts with a box size of 128 pixels at a down-sampled pixel size of 4.12 Å and subjected to a consensus 3D auto-refinement. After this consensus refinement, a mask containing only density for the bound ABD and the additional density was used for an initial round of 3D classification with 14 classes and no image alignment. Classes with consistent density were selected and

subjected to 3D auto-refinement. The resultant map was used as an updated reference for another round of 3D classification of the original 322,743 segment images with 6 classes, using an updated mask. After selecting 147,851 segment images from two classes with similarly placed density, a local 3D auto-refinement was performed, which showed substantially increased density in the masked region beyond the bound ABD. Using an updated mask, another round of 3D classification was run with 3 classes and no image alignment. One class, composed of 50,077 particles, with good density was selected. These particles were re-extracted at a box size of 384 pixels without down-sampling and locally refined using a 70 % z mask. These particles were then re-extracted with the plastin molecule at the center of the box and re-refined with a 50 % z mask. A subsequent round of 3D classification with 3 classes and no image alignment and was performed, and the best two classes were selected with 31,413 particles. After a local 3D auto-refinement, the particles were re-centered on the actin filament to optimize CTF refinement and Bayesian Polishing performance for two rounds. During the second round of particle polishing, the particles were once again re-centered on the bound T-plastin protein. Polished particles were subjected to a final 3D auto-refinement, which yielded a map assessed at 4.4 Å resolution. A final, local 3D auto-refinement was performed by masking only the plastin and two actin protomers to give the final map at 6.9 Å resolution (*SI Appendix*, Fig. S6) within this masked region.

Measurement of inter-filament distances

To provide reasonable inter-filament distances for the synthetic particles used to train the neural network, the distances between obvious filament bundles lying perpendicular to the electron beam were measured. Specifically, 98 micrographs were selected, down-sampled by 4, and low pass–filtered to 25 Å. One to four filament bundles were analyzed per micrograph (total of 127 filament pairs), by drawing a line through the central axis of one filament and drawing a parallel line through the axis of the other filament in the bundle using FIJI (*9*). The distance was measured based on the system of equations: $y1 = m*x + b1$; $y_2 = m*x + b_2$; $d = |b_2-b_1|/ \sqrt{(m^2 + 1)}$, where m is the shared slope between the two parallel lines, b1 and b2 are the intercepts for the

lines, x and y are the coordinates along the lines, and d is the distance between the two lines. These measurements revealed an average inter-filament distance of 159 Å with a standard deviation of 24 Å (*SI Appendix*, Fig S7B).

Synthetic dataset generation

We developed a convolutional neural network-based approach to specifically identify bundled filaments, while excluding individual filaments. To achieve full-micrograph segmentation, a neural network was first trained to recognize potential bundle configurations in synthetic data, and then used to predict on real data.

We first trained a denoising autoencoder on projections of plausible *in silico* bundle models and used the learned weights from this network to make a semantic segmentation network. The precise workflow for synthetic projection generation is outlined in *SI Appendix*, Fig. S8. Briefly, projection images were generated of zero to three filament units, with each filament unit consisting of either an individual filament or a two-filament bundle. To approximately reflect the frequency of bundles in the actual dataset, each filament unit had a 65% chance of being a bundle and 35% chance of being an individual filament. If the filament unit was a bundle, the filament would be copied, and the copy would be rotated about its helical axis by a random, uniformly sampled integer between 0° and 359°, randomly tilted by a bimodal Gaussian centered at 0° and 90°, with standard deviations of 1.5°, and then randomly translated in the y-direction by 159 Å ± 24 Å (empirically measured from 127 bundles in real micrographs), and uniformly translated in the z-direction (along the helical axis) by ±181 Å. Subsequently, for both individual and bundled filament units, the filament unit would be rotated about the phi and rot angles by a random, uniformly sampled value between 0° and 359°, and the tilt by 0° with a standard deviation of 2.5°. The filament unit was then randomly translated around the box by ±250 Å and projected along the z-axis to generate a noiseless projection. The same map was used to generate a noisy image paired with this noiseless projection, by adding pink noise in Fourier space, as implemented in EMAN2's python package to generate realistic-looking synthetic data (*10*). Three-channel stacks of semantic maps associated with the noisy/noiseless projection pairs

were generated by binarizing the filament unit and assigning it as a bundle or individual filament before projection.

Network architecture and training

A denoising autoencoder (DAE) was trained using the architecture outlined in *SI Appendix*, Fig. S9A. Each trainable layer had a ReLU activation function, except the final layer which had a linear activation function. The negative of the cross-correlation coefficient was used as the loss function. For training, the weights were initialized using the default initialization in TensorFlow. The model was trained using the Adam optimizer version of stochastic gradient descent with a learning rate of 0.00005 and minibatch size of 16 until the model converged (no improvement in validation loss for 3 epochs). Upon network convergence, the weights from the best epoch were restored. For training, 300,000 noisy / noiseless projection pairs with box sizes of 192 x 192 were generated, 90% of which was used for training and 10% for validation. Upon network convergence, the denoising autoencoder had an average cross-correlation coefficient of 0.984 on the validation set.

After training the model as a DAE, a semantic segmentation network was trained by copying all layers and weights of the DAE, except for the final layer, which was replaced with a 3-channel layer with softmax activation and default TensorFlow initialization. This semantic segmentation network was then trained with a learning rate of 0.00001. For training, 50,000 pairs of noisy inputs and semantically segmented targets of dimension 192 x 192 and 192 x 192 x 3, respectively, were used with a minibatch size of 16; 90% of the synthetic data was used for training and 10% for validation. The loss function was categorical cross-entropy, and upon network convergence the model had a categorical cross-entropy of 0.069 on the validation set. Example network performance on synthetic data is shown in *SI Appendix*, Fig. S9A,B.

Models were trained on a single NVIDIA 2080 Ti GPU with 11 GB of VRAM. Training required approximately 2.25 hours per epoch for the denoising autoencoder and 0.3 hours per epoch for the semantic segmentation network. Validation loss began to plateau around the fifth epoch for both models and then slowly improved until convergence (*SI Appendix*, Fig. S9C). As a

separate estimate of the 2D denoising reconstruction's resolution, 10,000 noiseless synthetic particle images not used during network training or validation were compared to noisy particle images denoised using the trained DAE, and the Fourier Ring Correlation (FRC) was computed (*SI Appendix*, Fig. S9D); the average FRC curve fell below 0.5 at 12.2 Å (~1.5 times Nyquist resolution), and 100 example FRC curves are also shown.

Particle picking using neural network

To pick particles, motion-corrected micrographs down-sampled by 4 were converted into semantic maps by extracting 192-pixel boxes across the micrograph in a raster pattern with 48 pixels of overlap and stitching back the output into a semantic map by computing a maximum intensity projection of the overlapping regions. Only the bundle channel results were used, and they were binarized using a fixed threshold of 0.85. After binarization, central axes of the bundles were selected for by excluding pixels near object borders using empirically derived parameters, and small objects were removed. The remaining binarized image was skeletonized, and non-maximum suppression was used to ensure all particle picks were spaced at least 148 Å away from each other. These particle coordinates were used for extraction in RELION.

Comparison to other particle pickers

To assess the capabilities of commonly used particle-picking software for the purpose of specifically picking bundles, we employed Topaz (*11*), crYOLO (*12*), and RELION's template-based auto-picker (*3*) on a subset of micrographs from the -Ca^{2+} dataset. Specifically, 50 micrographs with combinations of single- and multi-filament F-actin instances were selected; 25 micrographs were used for manual picking to train the networks, and 25 micrographs were used for assessment of picking quality and agreement with the neural network-based segmentation outlined in our work. A total of 3,377 particle coordinates of two-filament bundles, precisely centered, were hand-picked and used to train a crYOLO network using default parameters (PhosaurusNet architecture, batch size of 4, learning rate of 0.0001, trained for 200 epochs). After a network was trained, picking was performed on the test set of micrographs, and a

threshold of 0.25 was found to be optimal for picking performance. For Topaz training, the Topaz auto-picking feature as integrated into RELION4.0 (*13*) was used with the default parameters, except training was done for 15 epochs, instead of the default of 10, to improve results. After training, picking was performed on the test set of micrographs, and a -0.6 figure-of-merit threshold was found to be optimal for picking performance. Notably, in our experience, the crYOLO results had a large range of acceptable thresholds that produced similar results. Similarly, for our picker, a wide range of binarization thresholds applied to the semantically segmented map yielded very similar results because of the high network confidence. Conversely, for both Topaz and RELION's auto-picker, changing the threshold slightly drastically changed the number of picks, and we found the defaults had extremely high false-positive picks.

Particle picks from the various software were compared by visual inspection and quantitative comparison to the semantic segmentation from our neural network (*SI Appendix*, Fig. S11). Picks were visually assessed for their accuracy in centering the particle picks, and ability to discriminate single- from multi-filament bundles (*SI Appendix*, Fig. S11A). To assess agreement with our network's semantic segmentation of micrographs in the test set, picks were categorized based on which semantic bin the coordinate was positioned. For example, a picked coordinate that was in the bundle semantic channel was counted as a "true positive" in this case. Whereas a coordinate positioned on a pixel identified by the neural network as a single-filament was counted as a single filament pick, and the remaining picks were scored as background picks. Notably, the semantic maps are intentionally eroded to allow for good centering in our software, so many close, but un-centered picks were marked as background in this analysis (intentionally to account for pick centering).

Bundle processing

Visual inspection revealed that nearly all extracted particles had multiple filaments, but reference-free 2D classification with standard parameters produced classes with one filament or one well-resolved filament and one poorly resolved filament (*SI Appendix*, Fig. S7C). To prevent alignment from refining one filament at the expense of the other, particles images were extracted

in a large, 256-pixel box downsampled to 4.12 Å pixel size, and multiple rounds of 2D classification were performed in cryoSPARC (*14*), limiting the reconstruction resolution to 45 Å and the alignment resolution to 50 Å (*SI Appendix*, Fig. S12A). The "Align filament classes vertically" option was used to determine in-plane rotation, and at each iteration, 2D class averages were re-centered using a binary mask with a low threshold to maintain the bundle in the class average center. Particles were re-extracted with shifted, re-centered coordinates and psi angles were rotated by 90° for RELION helical conventions. With these re-centered particles, 2D classification with small translations yielded high-quality, reference-free 2D class averages in RELION, where obvious parallel and antiparallel 2D classes were present (*SI Appendix*, Fig. S12B). Despite exhaustive attempts at generating reasonable initial models using *ab initio* model generation, models would frequently be produced with one filament centered in the box or two poorly defined filaments.

Therefore, using the reference-free 2D class averages, initial models were generated using a custom projection-matching scheme. The map derived from single-filament helical analysis was rescaled and roughly positioned in the box to project onto one of the two filaments, then EMAN2's e2classvsproj script (*10*) was used on each filament to globally search Euler angles (with 10 degree sampling) and shifts (maximum of 84 Å) for an initial projection-match to the 2D class average. Finally, a custom projection-matching script using the EMAN2 python package was used to perform a finer, gradient-descent-based projection matching of each filament. While these projections had excellent correspondence to the 2D class averages, they did not have full 3D information to properly position filaments for an initial model. To properly z-position the filaments, a parallax-based approach using 2D classes that had clear side-views was used, and adjustments to the relative z-positions of the oriented filament maps was performed to maximize the cross-correlation between the side-view 2D class averages and the 3D models (*SI Appendix*, Fig. S12C). These maps were then lowpass filtered to 20 Å and used as initial references for 3D classification (*SI Appendix*, Fig. S12D).

3D classification required careful angular and translational searches to prevent head-on two-filament images from shifting to side-on views of one filament. An approach similar to

previous 3D classification schemes of large, mixed-population filamentous structures was used (*15*). Briefly, the tilt prior was kept fixed at 90° and global searches of rot, local, bimodal searches of psi, and local searches of tilt with 3.7° sampling and a 20° search range were performed, with a translation search range of 41.2 Å. After one iteration with these parameters, the 3D classification was interrupted; the tilt prior was updated, and translations were deleted. The 3D classification then resumed for three iterations, with global searches of rot, local, bimodal searches of psi, and local searches of tilt with a fine 1.8° sampling and 5° search range, and a large 123.6 Å translational search range was used.

During this supervised 3D classification, there was an apparent preferred orientation for particular rot angles (*SI Appendix*, Fig. S13A). 2D classification of particles with rot angles of 0±15° or 180±15° revealed that some side-on view 2D class averages look similar to individual filaments, and their constituent particle images were either bundled particles shifted to have one filament in the box center or poorly picked individual filaments. 2D classes with one centered filament that was much better resolved than other 2D classes were excluded and the angular distribution improved (*SI Appendix*, Fig. S13A,B). After this winnowing, five more iterations of 3D classification were performed with a smaller translational search of 20.6 Å, leading to 87,980 particles sorted into the parallel class and 69,408 particles sorted into the antiparallel class (*SI Appendix*, Fig. S13C). A 45% z-mask was generated for both classes, and subsequent signal subtraction and focused 3D classification without alignment removed particles without clear bridging plastin density. Un-subtracted particles were re-substituted, and following a consensus 3D auto-refinement, particles overlapping within the masked region were removed. A final, asymmetric, consensus 3D auto-refinement was performed on 41,701 particles for the parallel class, which reached 9.0 Å, and 28,759 particles for the antiparallel class, which was assessed at 10.0 Å (*SI Appendix*, Fig. S13D).

Variability analysis

During 3D auto-refinement, it became apparent that filament density quickly smeared outside the masked region, presumably due to the relative flexibility of the complex. This

observation, coupled with the composition of the system being two rigid bodies connected by a flexible cross-linking protein, led us to employ multi-body refinement, as implemented in RELION, to handle flexural heterogeneity (*16*). Using default 3D multi-body refinement parameters, with masks shown in *SI Appendix*, Fig. S13C, the resolution of the constituent filaments improved. Furthermore, we reasoned we could utilize the multi-body refinement parameters to measure the relative motions of the two filaments to each other. Specifically, volumes were generated for each particle image using RELION's relion_flex_analyze script (41,701 volumes for parallel, and 28,759 volumes for antiparallel), and atomic models of two separate plastin-decorated actin filaments were procedurally rigid-body docked into each of the volumes using scripting functions in UCSF Chimera (*17*). Distances and measurements between these docked models were measured using custom scripts employing functions from the ProDy package (*18*). Plots were generated with GraphPad Prism (Fig. 3E-H).

Model building and refinement

To generate homology models of the actin-binding 'core' (Fig. 1B) of plastin, the Robetta server was used (*19*). The selected homology model for 'prebound' T-plastin was the model that had the highest score. Sharpened, local-resolution-filtered maps as described above were used for model building. The high-resolution density maps were of sufficient quality for *de novo* atomic model building. As structures of components were available, initial models of actin (PDB 3j8a) and the 'prebound' T-plastin homology model were fit into the density map using Rosetta (*20*). Models were subsequently inspected and adjusted with Coot (*21*, *22*), and regions that underwent significant conformational rearrangements were manually rebuilt. The models were then subjected to several rounds of simulated annealing followed by real-space refinement in Phenix (*23*, *24*) alternating with manual adjustment in Coot. A final round of real-space refinement was performed without simulated annealing.

The pseudo-atomic models for the T-plastin prebundling state (Fig. 2) and both T-plastin bundle configurations (Fig. 3) were generated by rigid-body docking the high-resolution postbound T-plastin model and ABD1 fragments from the 'prebound' homology model, followed

by flexible fitting with ISOLDE (*25*), then real-space refinement in Phenix with harmonic restraints on the starting model enabled. Key statistics summarizing model building, refinement, and validation are reported in *SI Appendix*, Tables S1 and S2.

Structural figures and movies were prepared with ChimeraX (*26*). Structural superpositions were performed with the MatchMaker function in ChimeraX. Pruned RMSD, which excludes poorly aligning regions such as flexible loops, was calculated with the default parameters. Per-residue RMSD analysis was performed with UCSF Chimera (*17*) as previously described (*27*). The surface area of actin-binding interfaces was calculated with PDBePISA (*28*) (EMBL-EBI). Model quality was assessed with EMRinger (*29*) and MolProbity (*30*) as implemented in Phenix.

Sequence alignments

Protein sequences of human I-plastin (Q14651), human L-plastin (P13796), human T-plastin (P13797) were aligned with ClustalOmega (*31*) (EMBL-EBI).

Actin co-sedimentation assays

Mixtures of F-actin (5 μM) and the indicated T-plastin constructs (2 μM) were incubated at room temperature for 30 min in co-sedimentation buffer (10 mM Tris pH 7.5, 100 mM KCl, 2.5 mM $MgCl_2$, and 2 mM DTT. The samples were then spun for 30 min. at 16,000 rpm (low-speed) in a TLA-100 rotor and polycarbonate centrifugation tubes (Beckman Coulter No. 343775). This pellet was the ‘low-speed’ pellet. The supernatant was taken out from the centrifugation tube and then spun in a fresh centrifugation tube for another 30 min, at 100,000 rpm (high-speed). This pellet was the ‘high-speed’ pellet, and the supernatant was also collected. All three fractions were subject to SDS-PAGE and Coomassie staining. The gels were scanned using LI-COR imaging system, and subsequently quantified with ImageJ. Plots were generated with GraphPad Prism, and statistical analysis was performed with Microsoft Excel.

Cell culture and transfections

All experiments using HUVEC were performed with an hTERT-immortalized HUVEC line previously described (*32*) and cultured in EGM-2 media (Lonza, CC-3162). Cultures were regularly monitored for mycoplasma contamination via a PCR assay. For transient DNA transfections, 1.5×10^4 HUVEC, were plated the day before transfection in a glass-bottom 96-well plate (Cellvis) coated with 31 μg/mL collagen (Advanced BioMatrix, 5005-100 ML). The day of transfection, culture medium was replaced with 80 μL of antibiotics-free culture media per well. Then, 0.2 μg DNA encoding GFP-tagged T-Plastin (WT or mutant) and 0.25 μL Lipofectamine2000 (Thermo Fisher Scientific), mixed in 20 μL OptiMEM (Thermo Fisher Scientific), was added following the manufacturer's instructions. This transfection mix was replaced after 3 h with culture media and cells were processed for imaging after 24 h.

Fixed fluorescence imaging of cells

For fixed imaging, cells were rapidly fixed in 37 °C 4% paraformaldehyde in PBS, washed in PBS, permeabilized in 0.2% TritonX-100 in PBS, washed again and blocked in 3% FBS in PBS prior to primary and secondary antibody staining. Phalloidin conjugated to Alexa Fluor 568 (A12380, used at 1:500) and a secondary antibody against mouse IgG conjugated to Alexa Fluor 647 (A21235, used at 1:500) were from Thermo Fisher Scientific. The mouse monoclonal antibody against Arp3 (A5979, used at 1:250) was from Sigma.

All images were acquired on an automated confocal system controlled by Slidebook software (Intelligent Imaging Innovations, 3i). The system consists of an Eclipse-Ti body with perfect focus system (Nikon Instruments), CSU-W1 spinning disc (Yokogawa), a 3i laser stack with 405, 445, 488, 515, 561, and 640 nm lines, an environmental chamber (Haison), a 60X 1.27 NA water immersion objective, and two Zyla 4.2 sCMOS cameras (Andor) with motorized dichroics enabling simultaneous acquisition of channels.

FRAP assays and quantification

For live imaging, cell culture media was replaced with extracellular buffer (ECB) consisting of 125 mM NaCl, 5 mM KCl, 1.5 mM $MgCl_2$, 1.5 mM $CaCl_2$, 20 mM HEPES, supplemented with 10 mM D-glucose, 1% FBS, and 5 ng/mL bFGF (R&D Systems, 223-FB). To avoid interference from lamellipodial protrusion/retraction cycles on T-Plastin recovery measurements, HUVEC were first treated with 20 µM Y-27632 for 20 min., then a cocktail of 20 µM Y-27632, 8 µM Jasplakinolide, and 5 µM Latrunculin-B (JLY) for 30 min. to freeze actin turnover (*33*). Regions representing protrusions containing GFP-tagged T-plastin were selected for photobleaching with high intensity 488 nm laser light (Vector, Intelligent Imaging Innovations) and imaged every second for at least 3 minutes.

Analysis regions were subsequently defined in Slidebook to exclude the ends of actin stress fibers and focal adhesions, and the region intensities were measured to quantify their recovery after photobleaching. Intensities were background subtracted and normalized to their pre-bleach intensity, then analyzed in GraphPad Prism by fitting to an exponential one-phase association model, $Y = Y_0 + (Plateau - Y_0) * (1 - e^{(-K * x)})$. Wild-type and mutant data were compared using a two-way ANOVA with a mixed-effects model and Geisser-Greenhouse correction (*SI Appendix*, Table S3).

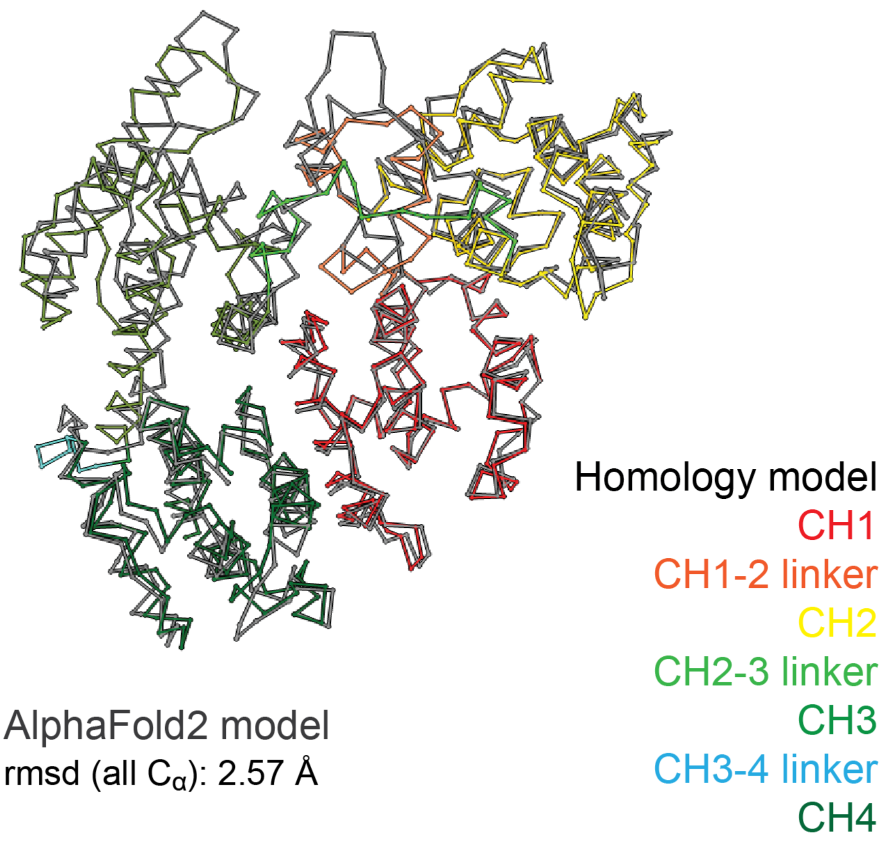

Fig. S1: Superposition of the prebound T-plastin homology model with the AlphaFold2 model of T-plastin (AF-P13797). The homology model is colored as indicated, and the AlphaFold2 model is displayed in grey.

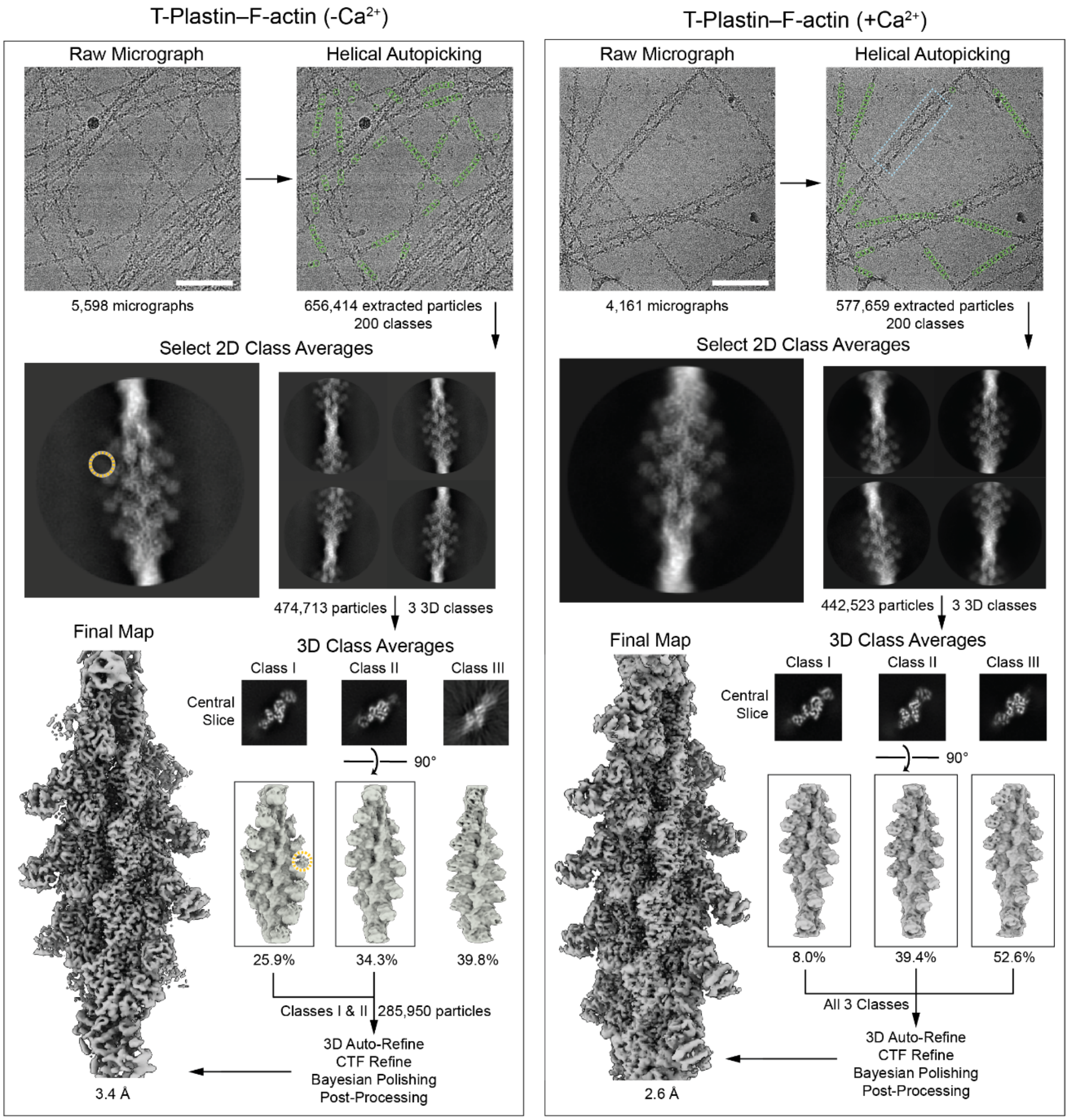

Fig. S2: Cryo-EM data processing workflow for single-filament T-plastin–F-actin complexes. Single-filament single particle helical cryo-EM processing workflow for T-plastin decorated F-actin datasets in the absence (left) and presence (right) of calcium. Scale bars, 100 nm. Yellow circles: weak signals in 2D and 3D classes of the –Ca^{2+} dataset targeted for subsequent focused classification. Blue box: a representative 2-filament F-actin bundle.

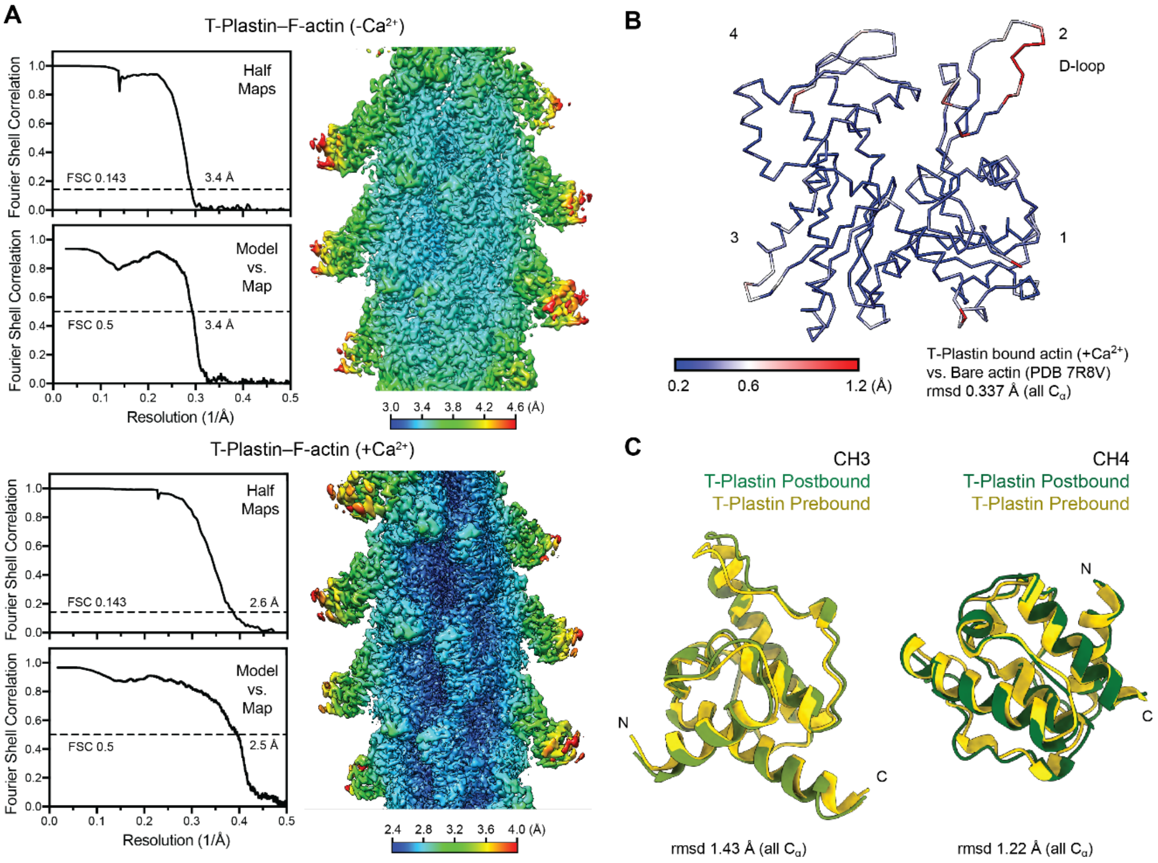

Fig. S3: Resolution assessment and analysis of single-filament T-plastin–F-actin complexes. (**A**) 3D reconstruction and map-model analyses (half-map Fourier Shell Correlation (FSC), map-to-model FSC, and local resolution) for datasets in the absence (top) and the presence (bottom) of calcium. (**B**) Actin protomer in C_α representation, colored by per-residue RMSD between the T-plastin–F-actin $+Ca^{2+}$ structure (this work) and bare F-actin (PDB 7r8v). Numerals indicate actin subdomains. (**C**) Individual superpositions of the prebound and postbound CH3 and CH4 domains.

A

```
I-Plastin (PLS1) -MENSTTTISREELEELQEAFNKIDIDNSGYVSDYELQDLFKEASLPLPGYKVREIVEKI 59
L-Plastin (PLS2) ---MARGSVSDEEMMELREAFAKVDTDGNGYISFNELNDLFKAACLPLPGYRVREITENL 57
T-Plastin (PLS3) MDEMATTQISKDELDELKEAFAKVDLNSNGFICDYELHELFKEANMPLPGYKVREIIQKL 60
                      :    :* :*: **:*** *:* :..*::.  **::*** * :*****:**** :::

I-Plastin (PLS1) LSVADSNKDGKISFEEFVSLMQELKSKDISKTFRKIINKREGITAIGGTSTISSEGTQHS 119
L-Plastin (PLS2) MATGDLDQDGRISFDEFIKIFHGLKSTDVAKTFRKAINKKEGICAIGGTSEQSSVGTQHS 117
T-Plastin (PLS3) MLDGDRNKDGKISFDEFVYIFQEVKSSDIAKTFRKAINRKEGICALGGTSELSSEGTQHS 120
                 :  .* ::**:***:**: ::: :**.*::***** **::*** *:****  ** *****

I-Plastin (PLS1) YSEEEKVAFVNWINKALENDPDCKHLIPMNPNDDSLFKSLADGILLCKMINLSEPDTIDE 179
L-Plastin (PLS2) YSEEEKYAFVNWINKALENDPDCRHVIPMNPNTNDLFNAVGDGIVLCKMINLSVPDTIDE 177
T-Plastin (PLS3) YSEEEKYAFVNWINKALENDPDCRHVIPMNPNTDDLFKAVGDGIVLCKMINLSVPDTIDE 180
                 ****** ****************:*:******  :.**:::.***:******** ******

                                     CH1
I-Plastin (PLS1) RAINKKKLTPFTISENLNLALNSASAIGCTVVNIGASDLKEGKPHLVLGLLWQIIKVGLF 239
L-Plastin (PLS2) RTINKKKLTPFTIQENLNLALNSASAIGCHVVNIGAEDLKEGKPYLVLGLLWQVIKIGLF 237
T-Plastin (PLS3) RAINKKKLTPFIIQENLNLALNSASAIGCHVVNIGAEDLRAGKPHLVLGLLWQIIKIGLF 240
                 *:********* *.*************** ******.**: ***:********:**:***

                      CH1-2 linker
I-Plastin (PLS1) ADIEISRNEALIALLNEGEELEELMKLSPEELLLRWVNYHLTNAGWHTISNFSQDIKDSR 299
L-Plastin (PLS2) ADIELSRNEALIALLREGESLEDLMKLSPEELLLRWANYHLENAGCNKIGNFSTDIKDSK 297
T-Plastin (PLS3) ADIELSRNEALAALLRDGETLEELMKLSPEELLLRWANFHLENSGWQKINNFSADIKDSK 300
                 ****:****** ***.:** **:*************.*:** *:* :.*.*** *****:

                                     CH2
I-Plastin (PLS1) AYFHLLNQIAPKGGEDG-PAIAIDLSGINETNDLKRAGLMLQEADKLGCKQFVTPADVVS 358
L-Plastin (PLS2) AYYHLLEQVAPKGDEEGVPAVVIDMSGLREKDDIQRAECMLQQAERLGCRQFVTATDVVR 357
T-Plastin (PLS3) AYFHLLNQIAPKGQKEGEPRIDINMSGFNETDDLKRAESMLQQADKLGCRQFVTPADVVS 360
                 **:***:*:**** ::* * : *::**:.*.:*::**  ***:*::***:**** :***

                                CH2-3 linker
I-Plastin (PLS1) GNPKLNLAFVANLFNTYPCLHKPNNNDIDMNLLEGESKEERTFRNWMNSLGVNPYINHLY 418
L-Plastin (PLS2) GNPKLNLAFIANLFNRYPALHKPENQDIDWGALEGETREERTFRNWMNSLGVNPRVNHLY 417
T-Plastin (PLS3) GNPKLNLAFVANLFNKYPALTKPENQDIDWTLLEGETREERTFRNWMNSLGVNPHVNHLY 420
                 *********:***** **.* **:*:***   ****::****************** :****

                                     CH3
I-Plastin (PLS1) SDLADALVIFQLYEMIRVPVNWSHVNKPPYPALGGNMKKIENCNYAVELGKNKAKFSLVG 478
L-Plastin (PLS2) SDLSDALVIFQLYEKIKVPVDWNRVNKPPYPKLGGNMKKLENCNYAVELGKNQAKFSLVG 477
T-Plastin (PLS3) ADLQDALVILQLYERIKVPVDWSKVNKPPYPKLGANMKKLENCNYAVELGKHPAKFSLVG 480
                 :** *****:**** *:***:*.:******* **.****:***********: *******

                                        CH3-4 linker
I-Plastin (PLS1) IAGQDLNEGNSTLTLALVWQLMRRYTLNVLSDLGEGEKVNDEIIIKWVNQTLKSANKKTS 538
L-Plastin (PLS2) IGGQDLNEGNRTLTLALIWQLMRRYTLNILEEIGGGQKVNDDIIVNWVNETLREAKKSSS 537
T-Plastin (PLS3) IGGQDLNDGNQTLTLALVWQLMRRYTLNVLEDLGDGQKANDDIIVNWVNRTLSEAGKSTS 540
                 *.*****:** ******:**********:*.::* *:*.**:**::***.** .* *.:*

                                     CH4
I-Plastin (PLS1) ISSFKDKSISTSLPVLDLIDAIAPNAVRQEMIRRENLSDEDKLNNAKYAISVARKIGARI 598
L-Plastin (PLS2) ISSFKDPKISTSLPVLDLIDAIQPGSINYDLLKTENLNDDEKLNNAKYAISMARKIGARV 597
T-Plastin (PLS3) IQSFKDKTISSSLAVVDLIDAIQPGCINYDLVKSGNLTEDDKHNNAKYAVSMARRIGARV 600
                 *.**** .**:** *:****** *..:. ::::  **.:::* ******:*:**:****:

I-Plastin (PLS1) YALPDDLVEVKPKMVMTVFACLMGKGLNRIK 629
L-Plastin (PLS2) YALPEDLVEVNPKMVMTVFACLMGKGMKRV- 627
T-Plastin (PLS3) YALPEDLVEVKPKMVMTVFACLMGRGMKRV- 630
                 ****:*****:*************:*::*:
```

B

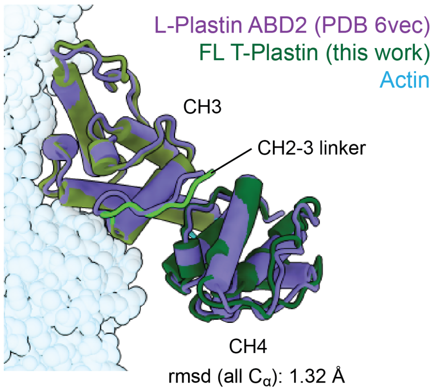

Fig. S4: Multiple sequence alignments and structural comparison of human plastin isoforms. (**A**) Multiple sequence alignment of human I-plastin (PLS1), L-plastin (LCP1/PLS2), and T-plastin (PLS3). (**B**) Superposition of F-actin bound L-Plastin ABD2 (PDB 6vec) and full-length T-plastin (this work).

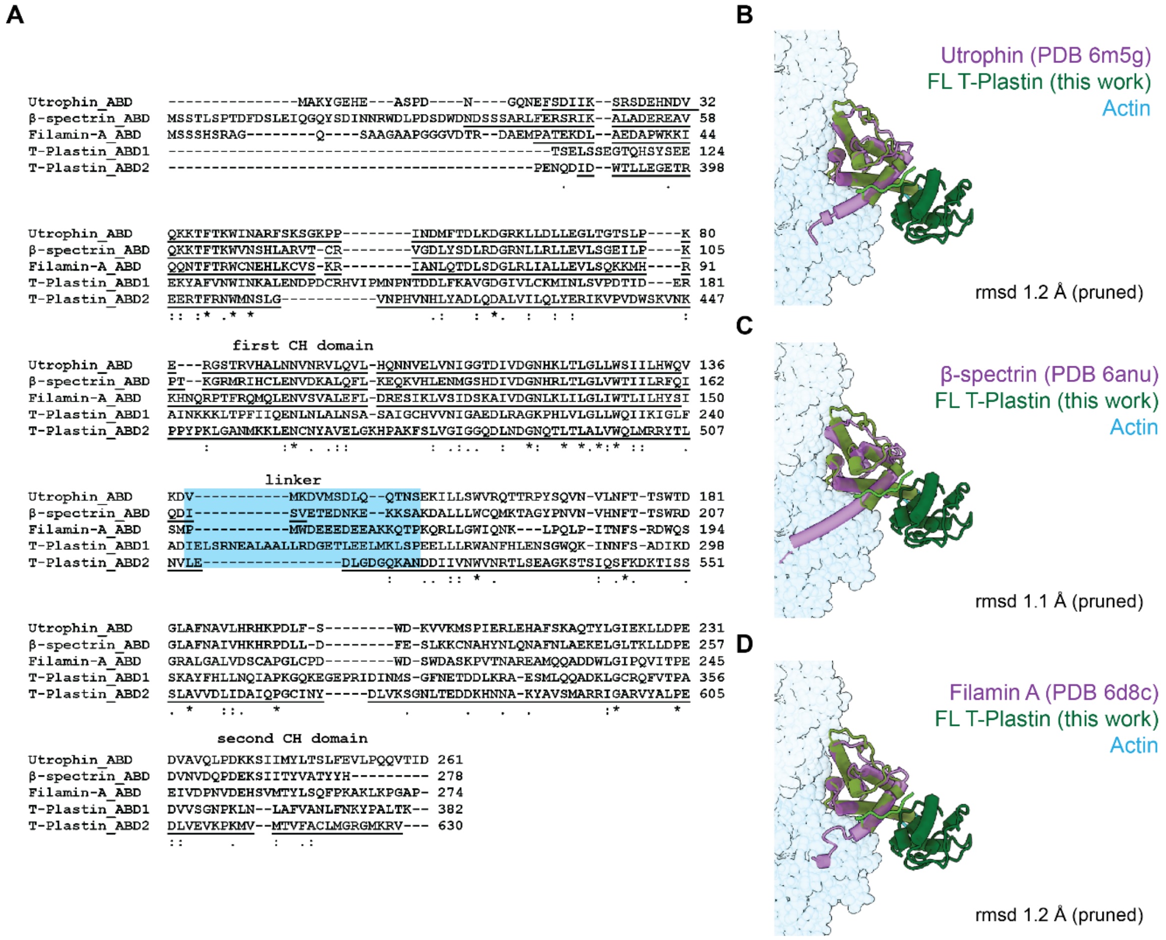

Fig. S5: Multiple sequence alignments and structural comparisons of actin-binding CH domains. (**A**) Multiple sequence alignment of the ABDs of human utrophin, β-spectrin, filamin A, and T-plastin. (**B-D**) Superpositions of F-actin bound utrophin ABD (PDB 6m5g, **B**), β-spectrin ABD (PDB 6anu, **C**), or filamin A ABD (PDB 6d8c, **D**) with T-plastin ABD2 (this work).

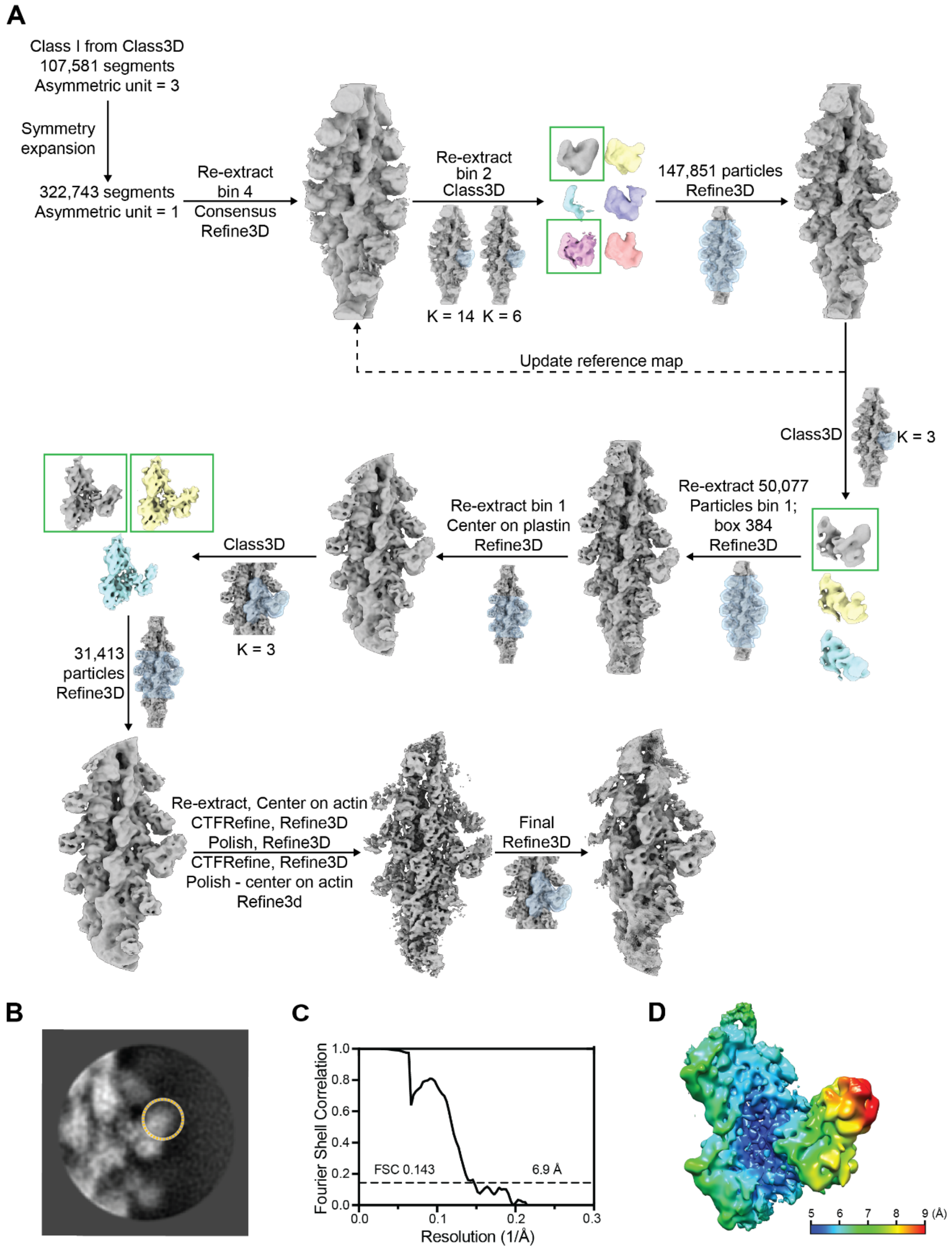

Fig. S6: Cryo-EM data processing workflow and data analysis for the prebundling state. (**A**) Cryo-EM data processing / focused classification workflow to visualize the prebundling state of the T-plastin–F-actin complex in the absence of calcium. (**B**) Representative 2D class average after masked 3D classification. Signal beyond ABD2 is highlighted in the yellow circle. (**C**) Half-map Fourier Shell Correlation (FSC) analysis of the final reconstruction. (**D**) Local resolution analysis of the final reconstruction.

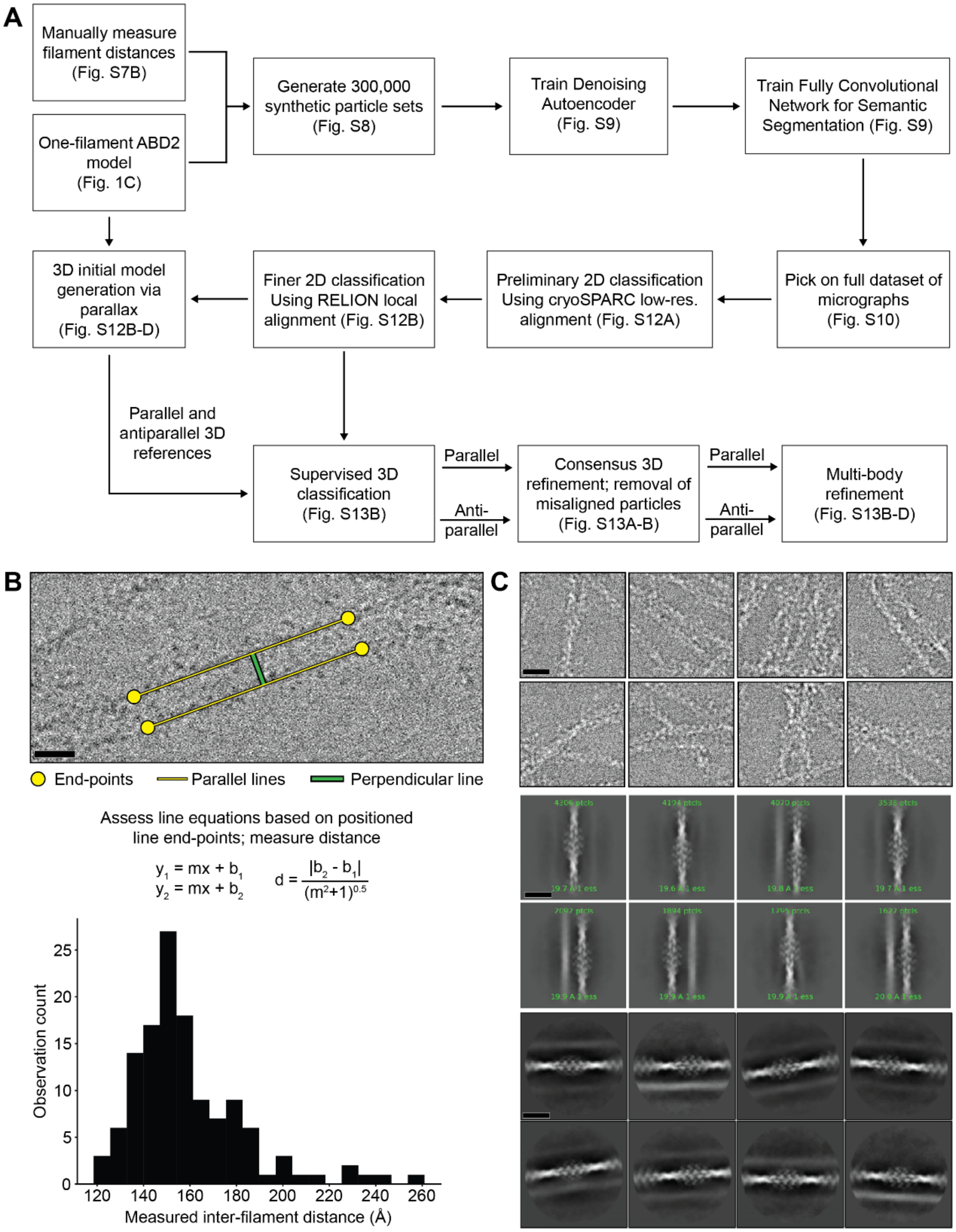

Fig. S7: Bundle processing workflow, manual distance measurements, and example 2D classes. (**A**) Overall workflow of plastin bundle processing. (**B**) Top: Distance measurement schematic drawn on representative 2-filament bundle. Scale bar, 200 Å. Two parallel line segments were traced through the filaments' axes (yellow line with ends as circles), and the distance between the two lines was calculated as illustrated. Bottom: Filament distance distribution from 127 measurements. (**C**) Top: Example extracted bundle segments (contrast inverted and low pass–filtered to 10 Å) containing multiple filaments. Example 2D classes with one clearly resolved filament and at least one poorly resolved filament from cryoSPARC (middle) and RELION (bottom) default 2D classification are displayed. Scale bars, 200 Å.

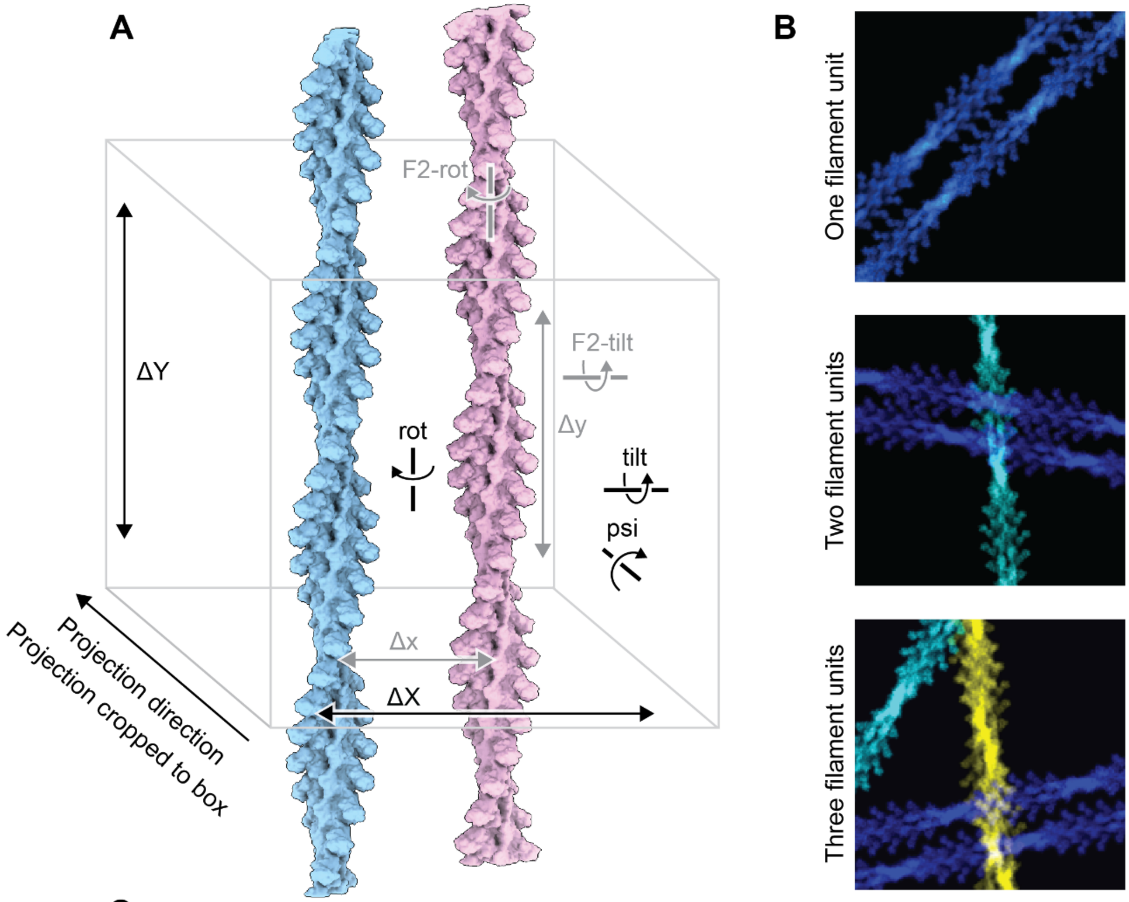

C

Symbol	Description	Sampling
	Number of filament units	Random integer [0,3], uniform distribution
	Probability of being bundle or single filament for each filament unit	65% chance of being a bundle, 35% chance of being a single filament
F2-rot	Rotation angle around central axis of the second filament if in a bundle unit	Random integer [0°,359°], uniform distribution
F2-tilt	Tilt angle of second filament if in a bundle unit	Bimodal distribution, 50% chance of parallel / antiparallel μ_p=0°, μ_{ap}=180°, σ=1.5°
Δx	x-translation of second filament	Normal distribution μ=159 Å, σ=24 Å
Δy	y-translation of second filament	Random real number [-181 Å,181 Å], uniform distribution
rot, psi	Rot and psi angle of whole filament unit	Random integer [0°,359°], uniform distribution
tilt	Tilt angle of whole filament unit	Normal distribution μ=0°, σ=2.5°
ΔX, ΔY	xy-translation of whole filament unit	Random real number [-250 Å, 250 Å], uniform distribution

Fig. S8: Generation of synthetic multi-bundle images for neural network training. (**A**) Diagram of 3D volume transformations applied to two T-plastin decorated filaments to generate plausible projection images of crosslinked actin filaments. (**B**) Pseudo-colored example synthetic projections with one (top), two (middle), and three (bottom) filament units. (**C**) Summary of parameter space sampled to generate synthetic datasets of plausible particle images of varying filament and bundle number.

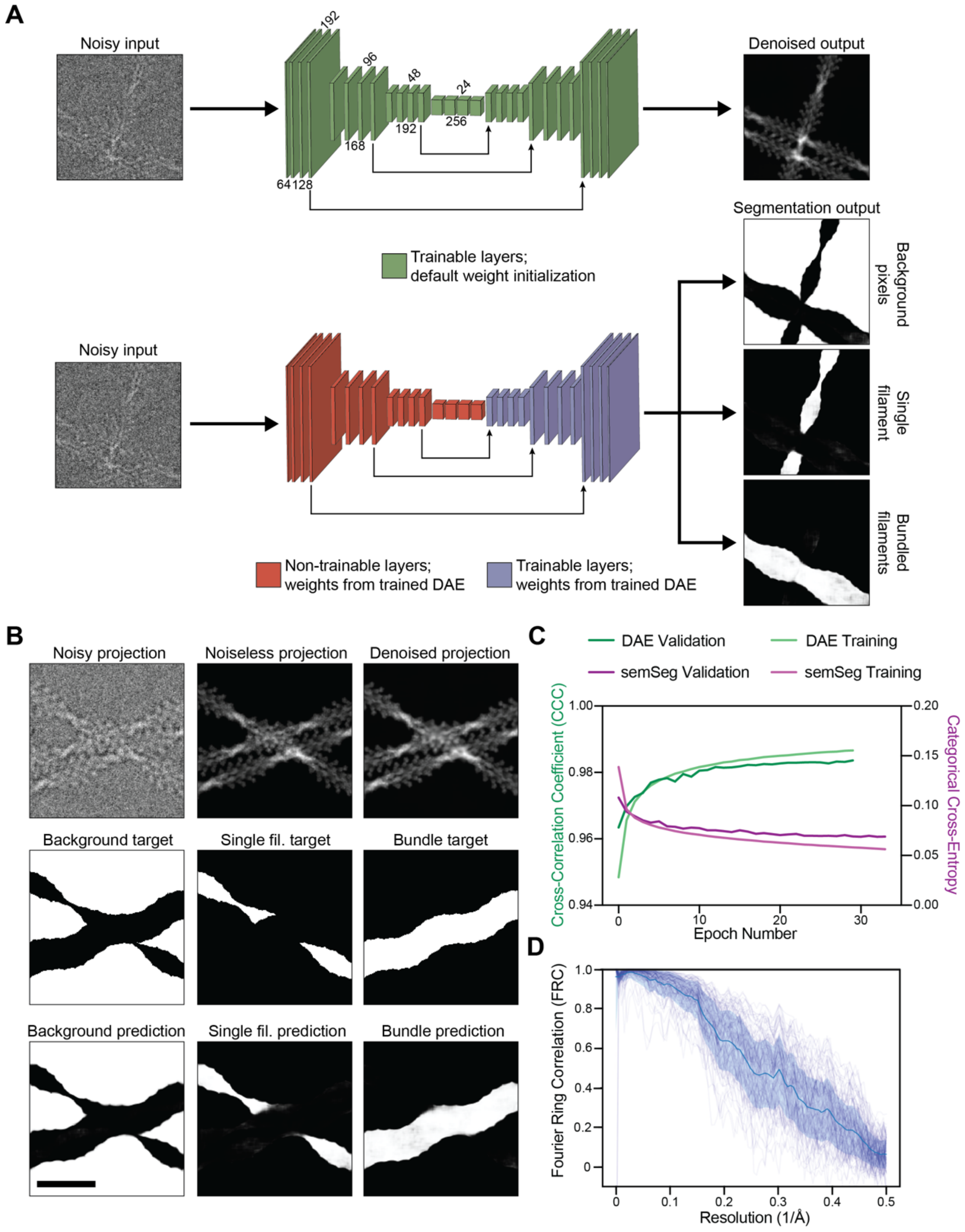

Fig. S9: Bundle picking neural network architecture and performance on synthetic datasets. (**A**) Schematic of data processing workflow for denoising autoencoder (top) and fully convolutional neural network for semantic segmentation (bottom). (**B**) Representative denoising and semantic segmentation performance on synthetic particle image containing one 2-filament bundle and one single filament. Scale bar, 30 nm. (**C**) Neural network training metrics across epochs. DAE: denoising auto-encoder; SemSeg: semantic segmentation. (**D**) Noiseless projection (ground truth)-to-denoised projection Fourier Ring Correlation (FRC) curves from 100 example images. Mean FRC +/- 1 S.D. computed from 10,000 randomly sampled images is shown in dark blue.

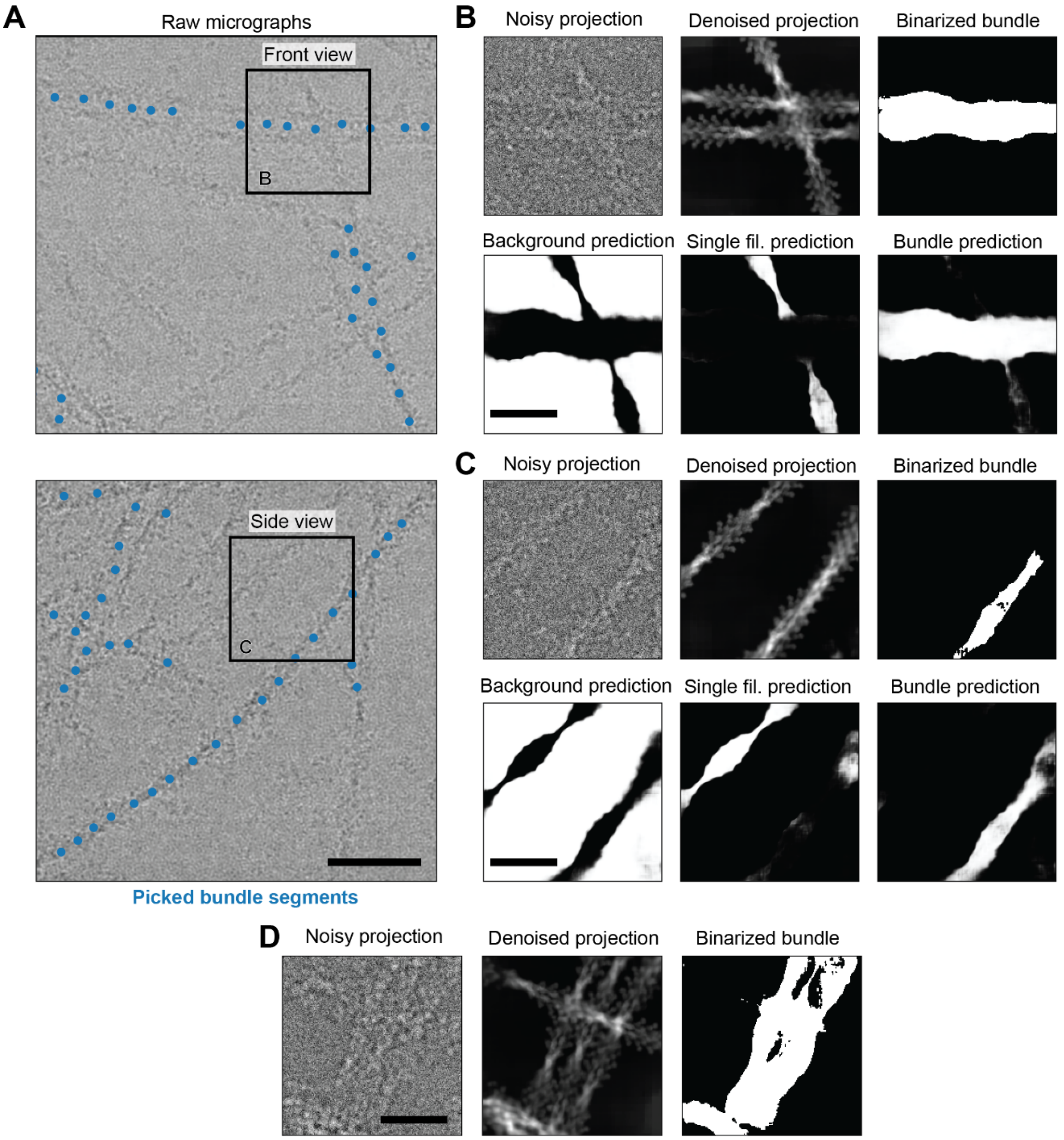

Fig. S10: Neural network performance on experimental cryo-EM data. (**A**) Example micrographs featuring single filaments and bundles, highlighting “railroad track” top view (top) and side view (bottom) of bundled filaments. Scale bar, 60 nm. (**B**) Network performance on top view particle from **A**. Top row, left: extracted particle image; middle: denoised particle image; right: binarized bundle channel used for particle picking. Bottom row, semantic segmentation channels denoting background, single filaments, and bundle. Scale bar, 30 nm. (**C**) Network performance on side view from **A**, which is not readily discriminated from single filaments by eye. Subpanels as in **B**, scale bar 30 nm**.** (**D**) Picked particle featuring a three-filament bundle, upon which the network was not explicitly trained. Scale bar, 30 nm.

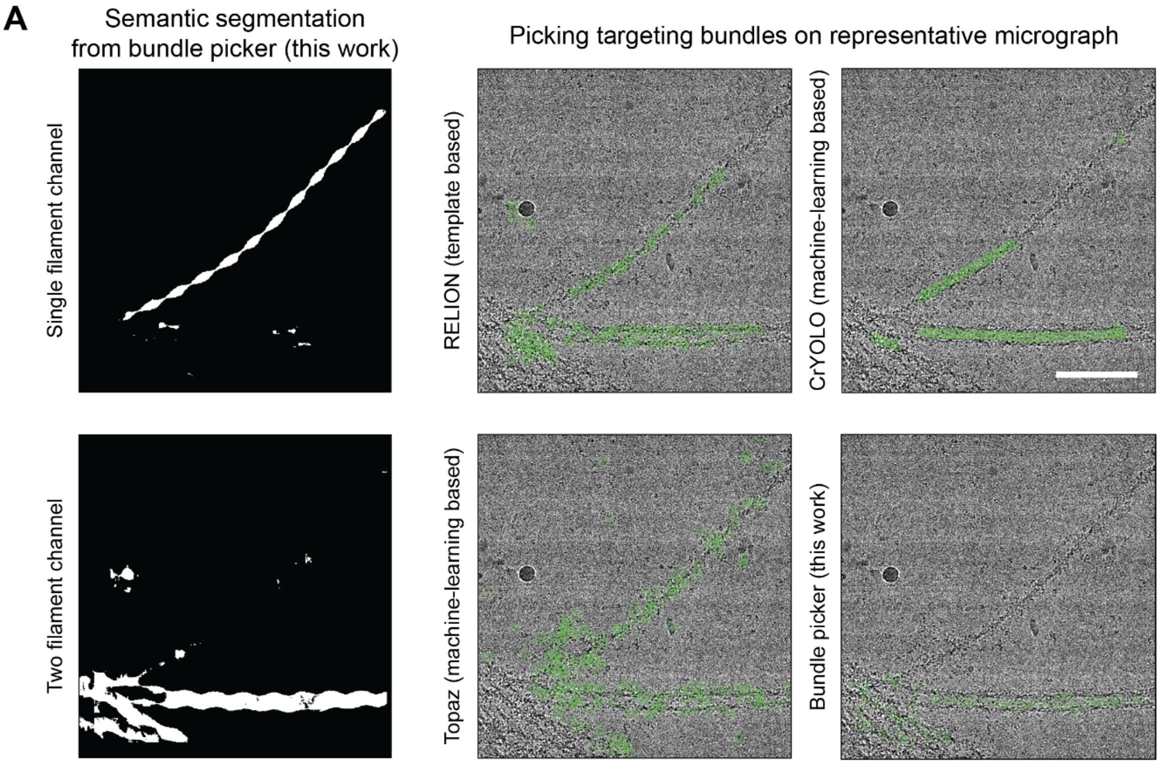

B

Picks overlapping with bundle picker's	RELION	Topaz	CrYOLO
Two filament channel (centered on either single filaments or bundles):	3,375 picks (61%)	1,164 picks (21%)	1,595 picks (64%)
One filament channel:	784 picks (14%)	341 picks (6%)	75 picks (3%)
Background channel:	1,329 picks (24%)	3,919 picks (72%)	807 picks (33%)

Fig. S11: Comparison of bundle detection between particle picking programs. (**A**) Left: Semantic segmentation maps of one- and two-filament channels generated by the neural network bundle picker presented in this work. Right: Picks (green circles) from indicated programs on a representative micrograph containing both single and bundled filaments. Scale bar, 100 nm. (**B**) Performance of other particle picking software compared to our bundle picker's semantic segmentation on a curated set of 25 micrographs.

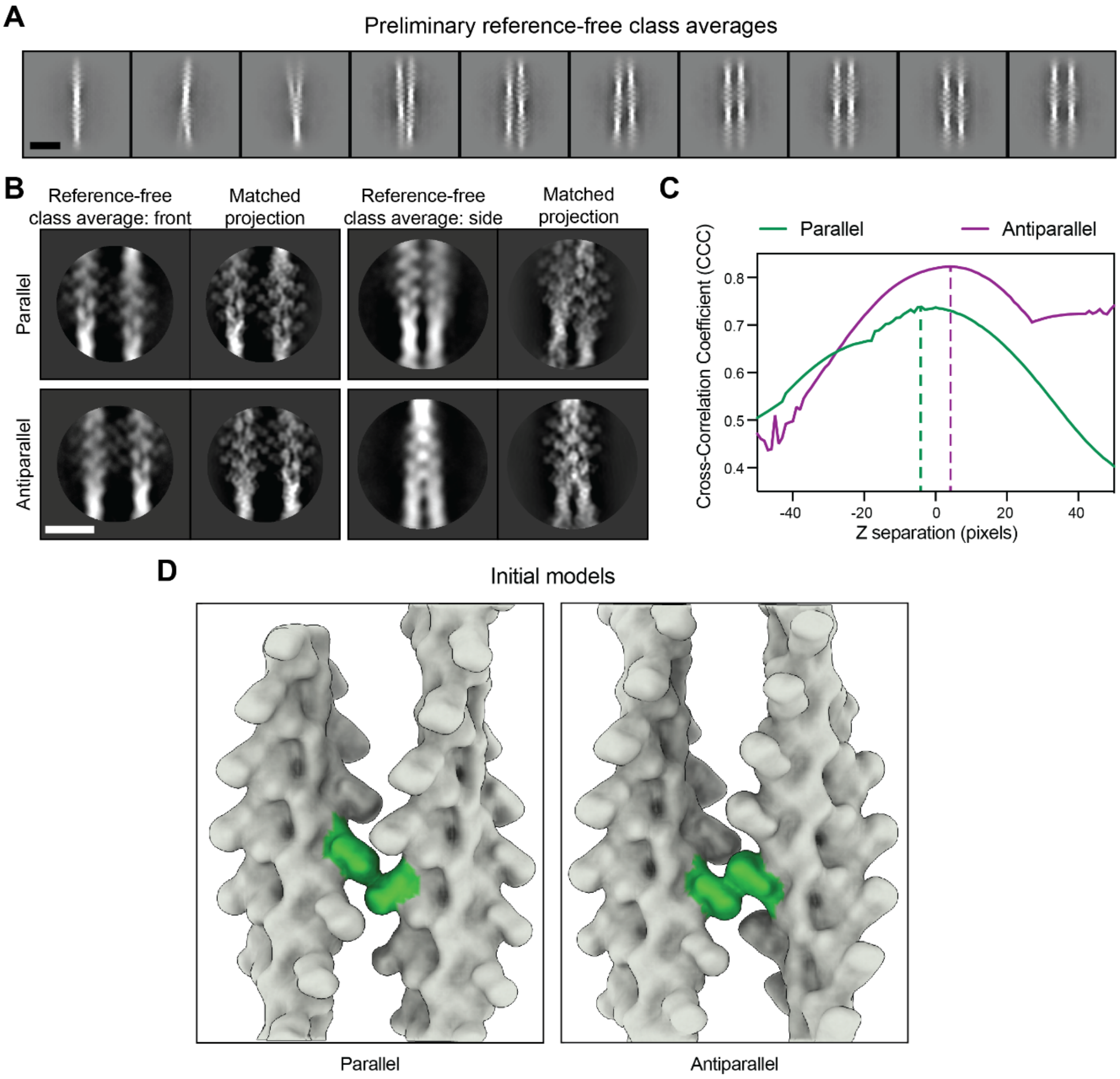

Fig. S12: Bundle cryo-EM 2D analysis and initial 3D model generation. (**A**) Reference-free 2D class averages generated with limited alignment and reconstruction resolutions to resolve both filaments. Scale bar, 30 nm. (**B**) Reference-free 2D class averages from high-resolution local alignment and corresponding matched projections of paired T-plastin decorated filaments positioned in 3D. Scale bar, 15 nm. (**C**) After initially positioning paired 3D volumes based on top view, cross-correlation was calculated between projections of models from side view as one filament is displaced in Z perpendicular to the top view plane and reference-free class averages corresponding to side view. The maximum cross-correlation coefficient is marked with a dashed line. (**D**) Initial 3D models for actin filaments bridged by T-plastin in parallel (left) and antiparallel (right) configurations. The cross-linking bridge is highlighted in green.

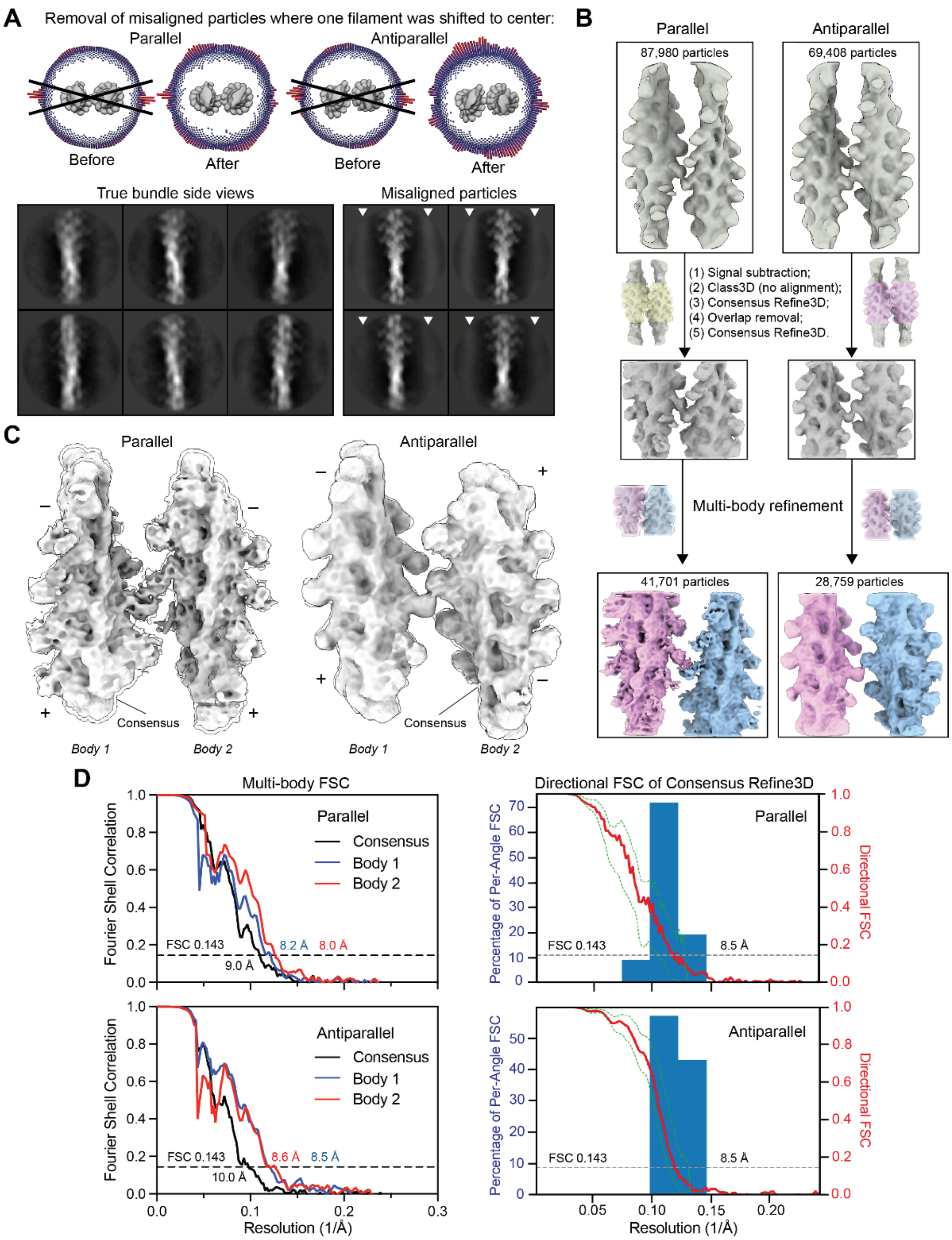

Fig. S13: Bundle cryo-EM 3D refinement workflow and resolution assessment. (**A**) 3D angular distributions of particle images prior to removing misaligned particles revealed an over-representation of side views inconsistent with their relatively low frequency in the raw data (top left). 2D classification of particles within the indicated angular wedge without alignment recovered true side views (bottom), as well as views with one filament centered surrounded by a misaligned haze (white arrowheads). Removing these misaligned particles substantially improved the 3D angular distributions (top right). (**B**) Multi-body refinement workflow. (**C**) Multibody reconstructions are displayed in grey, with corresponding consensus reconstructions shown as outline. (**D**) Resolution assessment of multi-body refinements (left) and directional Fourier Shell Correlation of the consensus Refine3D (right). Dashed green curves represent +/- S.D. from mean of directional FSC.

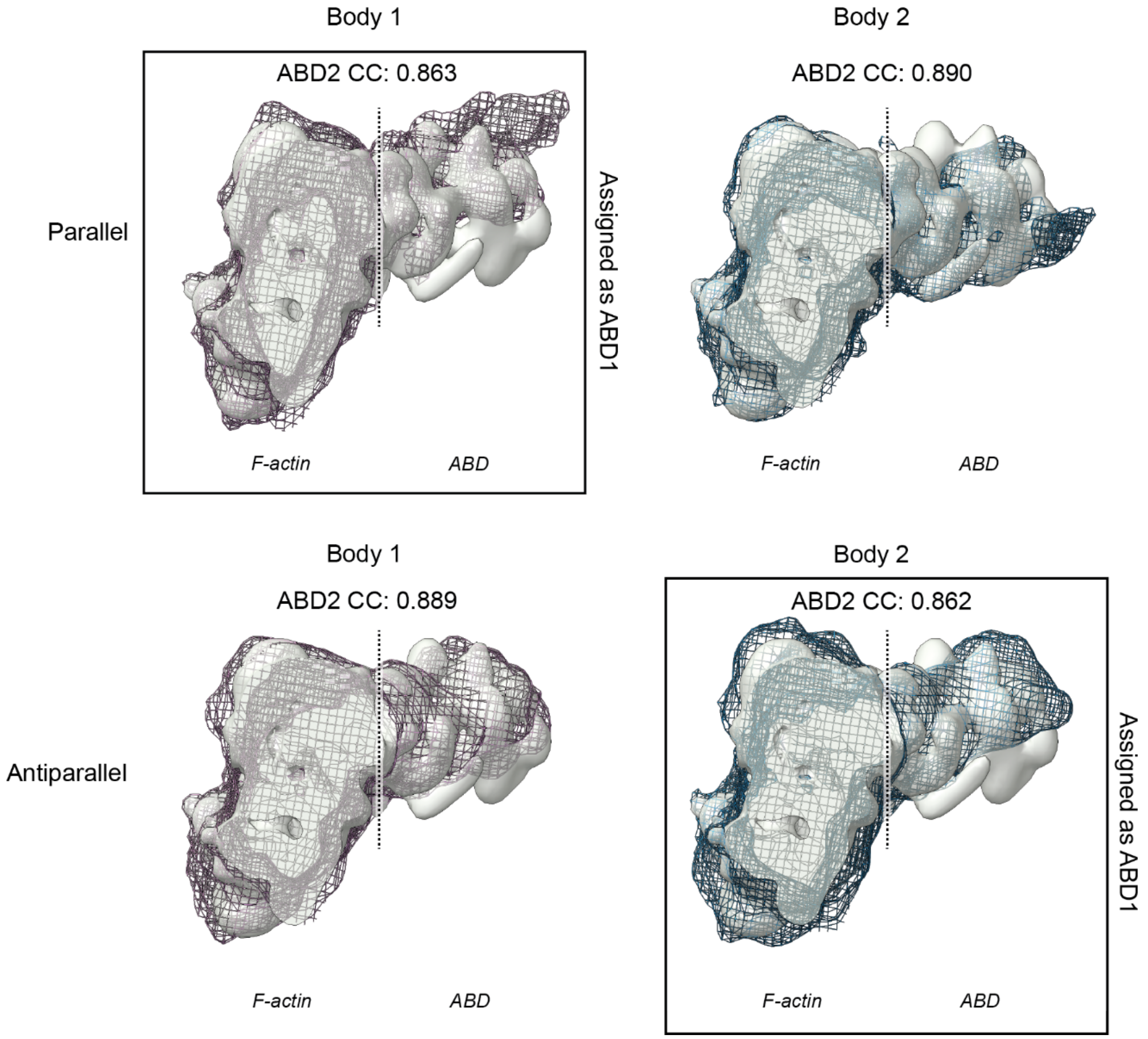

Fig. S14: Interpretation and ABD assignment of bundle reconstructions. Results of the indicated multi-body refinements are displayed in mesh, superimposed with the postbound reconstruction (where only ABD2 was resolved) low pass–filtered to 9 Å (transparent grey). Cross-correlation (CC) values were calculated by docking a 9 Å map simulated from the postbound atomic model (comprising ABD2 and 3 actin protomers) using the “Fit in map” function in UCSF Chimera.

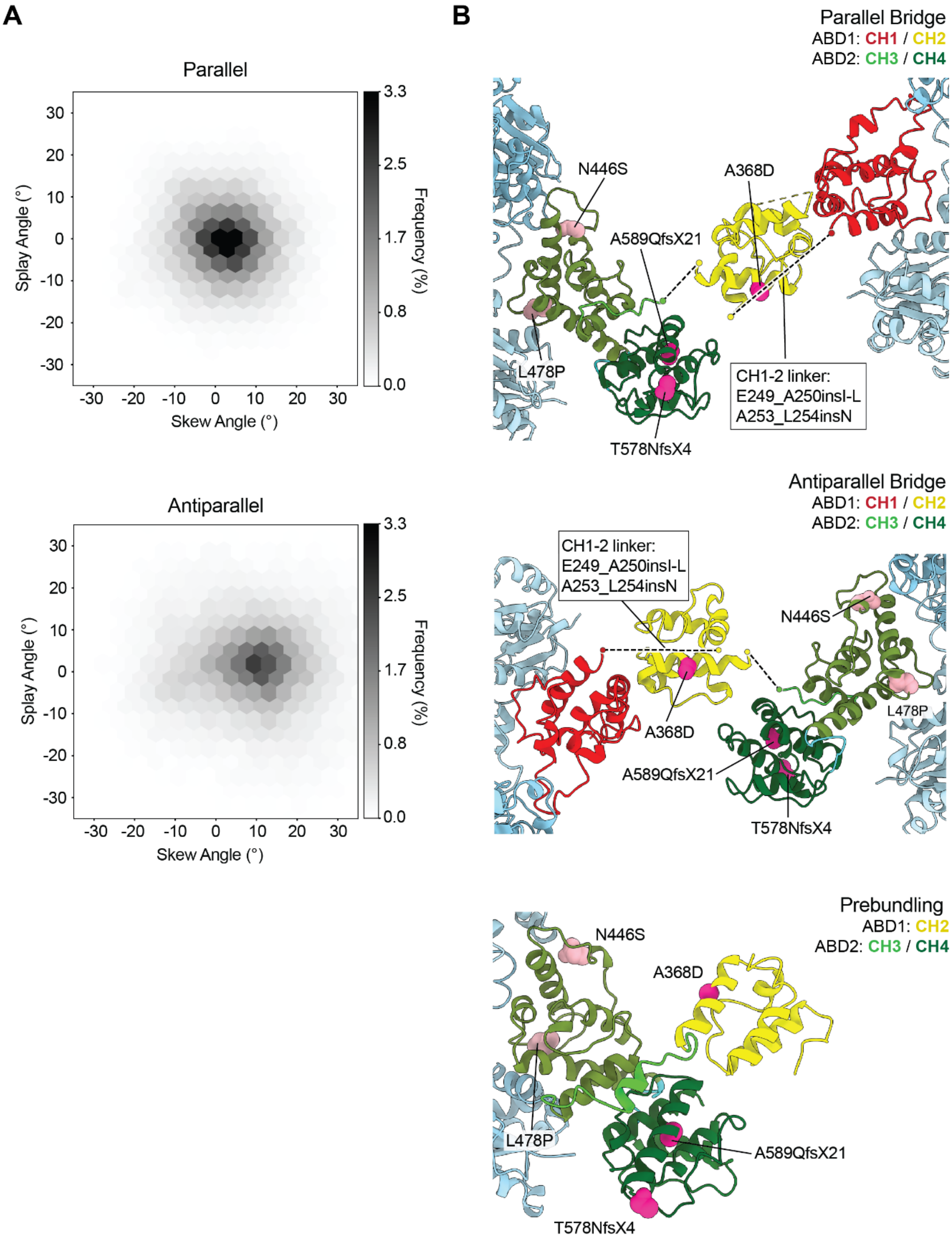

Fig. S15: Additional analysis of T-plastin rearrangements and mapping of disease mutations. (**A**) Replotting of measurements from **Fig. 3F**; there is no apparent coupling between skew and splay angles, suggesting T-plastin acts as a flexible joint within the allowable conformational space of each bridge configuration. (**B**) Osteoporosis-linked T-plastin mutations mapped onto the parallel bridge (top), the antiparallel bridge (middle), and the prebundling (bottom) states of T-plastin.

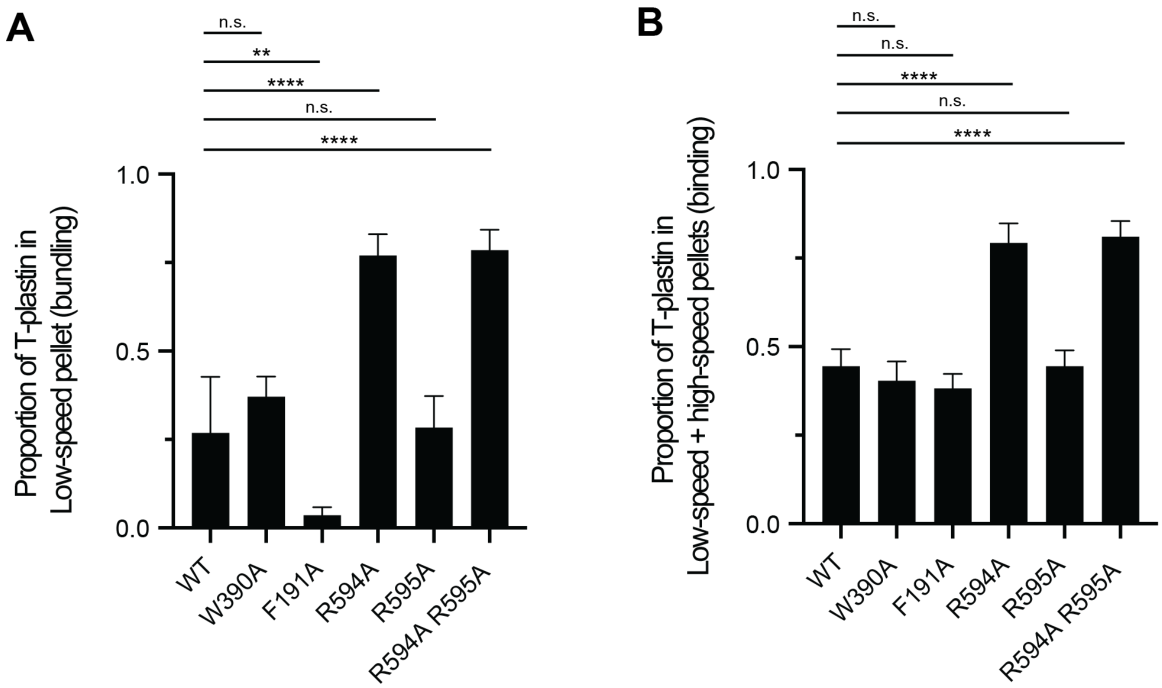

Fig. S16: Statistical analysis of co-sedimentation assays. Replotting of data from **Fig. 4C and Fig. S17** as individual bars for proportion of T-plastin in (**A**) low-speed pellet (WT / W390A: $p = 0.16$; WT / F191A: $p = 0.008$; WT / R594A: $p = 0.00006$; WT / R595A: $p = 0.85$; WT / R594A R595A: $p = 0.00005$) and (**B**) low-speed pellet + high-speed pellet (WT / W390A: $p = 0.26$; WT / F191A: $p = 0.06$; WT / R594A: $p = 0.00006$; WT / R595A: $p = 0.99$; WT / R594A R595A: $p = 0.00005$). WT: $n = 7$; mutants: $n = 4$. n.s.: not significant, $^{**}p < 0.01$, $^{****}p < 0.0001$, two-tailed T-test.

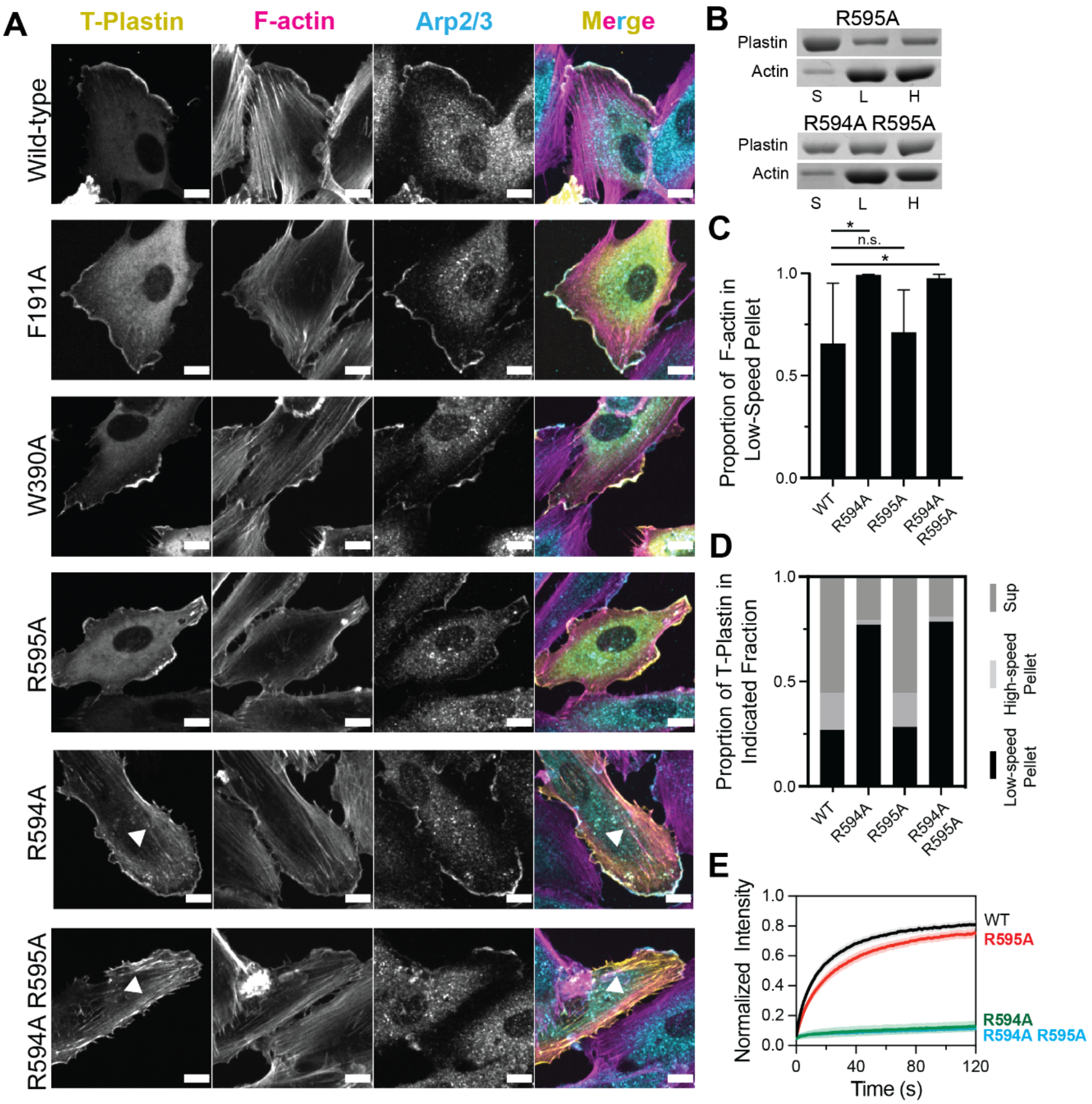

Fig. S17: Additional analysis of T-plastin mutants. (**A**) Fluorescence imaging of the indicated T-plastin constructs expressed in HUVEC cells. T-plastin: eGFP; Arp2/3 (marking branched actin at the leading edge): ARP3 immunofluorescence; F-actin: phalloidin. Scale bar, 10 μm. Arrowheads indicate stress fiber / focal adhesion localization in R594A and R594A R595A constructs (bottom 2 rows). (**B**) SDS-PAGE of low-speed / high-speed F-actin co-sedimentation assays with the indicated constructs. S, supernatant; L, low-speed pellet; H, high-speed pellet. (**C**) Quantification of **B**: proportion of F-actin in low-speed pellet (indicative of bundling). Error bars represent S.D. WT: n = 7; Mutants: n = 4. WT / R594A: $p = 0.03$; WT / R595A: $p = 0.74$; WT / R594A R595A: $p = 0.04$. n.s.: not significant, *$p < 0.05$, two-tailed T-test. Wild-type and R594A are replotted from **Fig. 4C**. (**D**) Quantification of **B**: proportions of indicated T-plastin constructs in each fraction. Wild-type and R594A are replotted from **Fig. 4D**. See **Fig. S16** for statistical analysis. (**E)** FRAP assays of the indicated eGFP tagged T-plastin constructs.

Table S1. High resolution cryo-EM data collection, refinement, and validation statistics.

	T-Plastin ABD–F-actin (+Ca^{2+}) (EMD-24323, PDB 7R94)	T-Plastin ABD–F-actin (–Ca^{2+}) (EMD-25496)
**Data collection and processing**		
Microscope	Titan Krios	Titan Krios
Voltage (kV)	300	300
Detector	K2 Summit	K2 Summit
Magnification	29,000	29,000
Electron exposure (e^{-}Å^{-2})	71	60
Exposure rate (e^{-}/pixel/s)	7.5	6
Calibrated pixel size (Å)	1.06	1.03
Defocus range (μm)	–1.5 to –3.5	–1.5 to –3.5
Symmetry imposed	C1	C1
	27.4 Å rise	27.0 Å rise
	–166.8° twist	–166.8° twist
Initial particle images (no.)	577,659	656,414
Final particle images (no.)	442,523	474,713
Map resolution (Å)	2.60	3.44
FSC threshold	0.143	0.143
**Refinement**		
Initial models (PDB ID)	7R8V, 1RT8, 1PXY	
Model resolution (Å)	2.52	3.39
FSC threshold	0.5	0.5
Map sharpening B factor (Å^2)	–42.3	–96.0
Model composition	5 actin protomers, 2 T-plastin actin-binding domains	
Non-hydrogen atoms	20,247	
Protein residues	2,570	
Ligands	5 Mg.ADP	
B factors (Å^2)		
Protein	52.08	
Ligand	35.46	
R.M.S. deviations		
Bond lengths (Å)	0.005	
Bond angles (°)	0.650	
**Validation**		
MolProbity score	1.50	
Clashscore	4.98	
Poor rotamers (%)	0.05	
Ramachandran plot		
Favored (%)	96.38	
Allowed (%)	3.62	
Disallowed (%)	0.00	
EMRinger Score	4.65	

Table S2. Medium resolution cryo-EM data collection, refinement, and validation statistics.

	T-Plastin ABD–F-actin, prebundling (EMD-25496, PDB 7SXA)	T-Plastin ABD–F-actin, parallel bundle (EMD-25494, PDB 7SX8)	T-Plastin ABD–F-actin, antiparallel bundle (EMD-25495, PDB 7SX9)
**Data collection and processing**			
Microscope	Titan Krios		
Voltage (kV)	300		
Detector	K2 Summit		
Magnification	29,000		
Electron exposure ($e^{-}Å^{-2}$)	60		
Exposure rate (e^{-}/pixel/s)	6		
Calibrated pixel size (Å)	1.03		
Defocus range (μm)	–1.5 to –3.5		
Symmetry imposed	C1		
Initial particle images (no.)	322,743	87,980	69,408
Final particle images (no.)	31,413	41,701	28,759
Map resolution (Å)	6.85	9.04 (consensus)<br>8.16 (body 1)<br>7.95 (body 2)	9.97 (consensus)<br>8.45 (body 1)<br>8.56 (body 2)
FSC threshold	0.143		
**Refinement**			
Initial models (PDB ID)	7R94 (this work), 1RT8, 1PXY		
Map sharpening B factor ($Å^2$)	–216.4	–600 (consensus)<br>–450 (body 1/2)	N.A. (consensus)<br>–300 (body 1/2)
Model composition	3 actin protomers, 1 T-plastin	6 actin protomers, 1 T-plastin	
Non-hydrogen atoms	11,272	21,036	21,036
Protein residues	1,431	2,668	2,668
Ligands	3 Mg.ADP	6 Mg.ADP	
R.M.S. deviations			
Bond lengths (Å)	0.007	0.002	0.004
Bond angles (°)	1.134	0.660	0.893
**Validation**			
MolProbity score	1.82	1.91	1.54
Clashscore	9.77	13.07	4.78
Poor rotamers (%)	0.99	0.18	1.06
Ramachandran plot			
Favored (%)	95.55	95.89	95.97
Allowed (%)	4.10	4.03	3.84
Disallowed (%)	0.35	0.08	0.19

Table 3. FRAP fitting parameters and analysis.

T-plastin construct	k (s^{-1})	$t_{1/2}$ (s)	Mobile fraction (%)	P value versus wild-type
Wild-type	0.04	17.7	82	-
F191A	0.07	8.8	87	0.0001
W390A	0.02	34.6	76	0.0144
R594A	0.003	212.9	25	< 0.0001
R595A	0.02	27.9	80	0.0048
R594A R595A	0.001	515.2	34	< 0.0001

Movie S1. Interpolation along the first principal component from the multi-body refinement of the parallel bundle. The measured splay and skew angles are displayed at each frame.

Movie S2. Interpolation along the first principal component from the multi-body refinement of the antiparallel bundle. The measured splay and skew angles are displayed at each frame.

Movie S3. Sequence of T-plastin conformational transitions resulting in parallel bundle. Colored as in Fig. 3A.

Movie S4. Sequence of T-plastin conformational transitions resulting in antiparallel bundle. Colored as in Fig. 3A.

SI References

1. P. S. Gurel, L. Y. Kim, P. V. Ruijgrok, T. Omabegho, Z. Bryant, G. M. Alushin, Cryo-EM structures reveal specialization at the myosin VI-actin interface and a mechanism of force sensitivity. *eLife*. **6**, e31125 (2017).

2. D. N. Mastronarde, Automated electron microscope tomography using robust prediction of specimen movements. *J Struct Biol*. **152**, 36–51 (2005).

3. J. Zivanov, T. Nakane, B. O. Forsberg, D. Kimanius, W. J. Hagen, E. Lindahl, S. H. Scheres, New tools for automated high-resolution cryo-EM structure determination in RELION-3. *eLife*. **7**, e42166 (2018).

4. L. Mei, S. Espinosa de los Reyes, M. J. Reynolds, R. Leicher, S. Liu, G. M. Alushin, Molecular mechanism for direct actin force-sensing by α-catenin. *eLife*. **9**, e62514 (2020).

5. S. Q. Zheng, E. Palovcak, J.-P. Armache, K. A. Verba, Y. Cheng, D. A. Agard, MotionCor2: anisotropic correction of beam-induced motion for improved cryo-electron microscopy. *Nat Methods*. **14**, 331–332 (2017).

6. J. Zivanov, T. Nakane, S. H. W. Scheres, A Bayesian approach to beam-induced motion correction in cryo-EM single-particle analysis. *IUCrJ*. **6**, 5–17 (2019).

7. A. Rohou, N. Grigorieff, CTFFIND4: Fast and accurate defocus estimation from electron micrographs. *J Struct Biol*. **192**, 216–221 (2015).

8. T. Grant, N. Grigorieff, Measuring the optimal exposure for single particle cryo-EM using a 2.6 Å reconstruction of rotavirus VP6. *Elife*. **4**, e06980 (2015).

9. J. Schindelin, I. Arganda-Carreras, E. Frise, V. Kaynig, M. Longair, T. Pietzsch, S. Preibisch, C. Rueden, S. Saalfeld, B. Schmid, J.-Y. Tinevez, D. J. White, V. Hartenstein, K. Eliceiri, P. Tomancak, A. Cardona, Fiji: an open-source platform for biological-image analysis. *Nat Methods*. **9**, 676–682 (2012).

10. G. Tang, L. Peng, P. R. Baldwin, D. S. Mann, W. Jiang, I. Rees, S. J. Ludtke, EMAN2: An extensible image processing suite for electron microscopy. *Journal of Structural Biology*. **157**, 38–46 (2007).

11. T. Bepler, A. Morin, M. Rapp, J. Brasch, L. Shapiro, A. J. Noble, B. Berger, Positive-unlabeled convolutional neural networks for particle picking in cryo-electron micrographs. *Nat Methods*. **16**, 1153–1160 (2019).

12. T. Wagner, L. Lusnig, S. Pospich, M. Stabrin, F. Schönfeld, S. Raunser, Two particle-picking procedures for filamentous proteins: SPHIRE-crYOLO filament mode and SPHIRE-STRIPER. *Acta Crystallogr D Struct Biol*. **76**, 613–620 (2020).

13. D. Kimanius, L. Dong, G. Sharov, T. Nakane, S. H. W. Scheres, New tools for automated cryo-EM single-particle analysis in RELION-4.0. *Biochem J*. **478**, 4169–4185 (2021).

14. A. Punjani, J. L. Rubinstein, D. J. Fleet, M. A. Brubaker, cryoSPARC: algorithms for rapid unsupervised cryo-EM structure determination. *Nat Methods*. **14**, 290–296 (2017).

15. A. D. Cook, S. W. Manka, S. Wang, C. A. Moores, J. Atherton, A microtubule RELION-based pipeline for cryo-EM image processing. *Journal of Structural Biology*. **209**, 107402 (2020).

16. T. Nakane, D. Kimanius, E. Lindahl, S. H. Scheres, Characterisation of molecular motions in cryo-EM single-particle data by multi-body refinement in RELION. *eLife*. **7**, e36861 (2018).

17. E. F. Pettersen, T. D. Goddard, C. C. Huang, G. S. Couch, D. M. Greenblatt, E. C. Meng, T. E. Ferrin, UCSF Chimera--A visualization system for exploratory research and analysis. *J. Comput. Chem.* **25**, 1605–1612 (2004).

18. A. Bakan, L. M. Meireles, I. Bahar, ProDy: Protein Dynamics Inferred from Theory and Experiments. *Bioinformatics*. **27**, 1575–1577 (2011).

19. D. E. Kim, D. Chivian, D. Baker, Protein structure prediction and analysis using the Robetta server. *Nucleic Acids Research*. **32**, W526–W531 (2004).

20. R. Y.-R. Wang, Y. Song, B. A. Barad, Y. Cheng, J. S. Fraser, F. DiMaio, Automated structure refinement of macromolecular assemblies from cryo-EM maps using Rosetta. *eLife*. **5**, e17219 (2016).

21. A. Brown, F. Long, R. A. Nicholls, J. Toots, P. Emsley, G. Murshudov, Tools for macromolecular model building and refinement into electron cryo-microscopy reconstructions. *Acta Crystallogr D Biol Crystallogr*. **71**, 136–153 (2015).

22. P. Emsley, B. Lohkamp, W. G. Scott, K. Cowtan, Features and development of *Coot*. *Acta Crystallogr D Biol Crystallogr*. **66**, 486–501 (2010).

23. P. D. Adams, P. V. Afonine, G. Bunkóczi, V. B. Chen, I. W. Davis, N. Echols, J. J. Headd, L.-W. Hung, G. J. Kapral, R. W. Grosse-Kunstleve, A. J. McCoy, N. W. Moriarty, R. Oeffner, R. J. Read, D. C. Richardson, J. S. Richardson, T. C. Terwilliger, P. H. Zwart, *PHENIX* : a comprehensive Python-based system for macromolecular structure solution. *Acta Crystallogr D Biol Crystallogr*. **66**, 213–221 (2010).

24. P. V. Afonine, B. K. Poon, R. J. Read, O. V. Sobolev, T. C. Terwilliger, A. Urzhumtsev, P. D. Adams, Real-space refinement in *PHENIX* for cryo-EM and crystallography. *Acta Crystallogr D Struct Biol*. **74**, 531–544 (2018).

25. T. I. Croll, *ISOLDE* : a physically realistic environment for model building into low-resolution electron-density maps. *Acta Crystallogr D Struct Biol*. **74**, 519–530 (2018).

26. T. D. Goddard, C. C. Huang, E. C. Meng, E. F. Pettersen, G. S. Couch, J. H. Morris, T. E. Ferrin, UCSF ChimeraX: Meeting modern challenges in visualization and analysis: UCSF ChimeraX Visualization System. *Protein Science*. **27**, 14–25 (2018).

27. R. Zhang, G. M. Alushin, A. Brown, E. Nogales, Mechanistic Origin of Microtubule Dynamic Instability and Its Modulation by EB Proteins. *Cell*. **162**, 849–859 (2015).

28. E. Krissinel, K. Henrick, Inference of Macromolecular Assemblies from Crystalline State. *Journal of Molecular Biology*. **372**, 774–797 (2007).

29. B. A. Barad, N. Echols, R. Y.-R. Wang, Y. Cheng, F. DiMaio, P. D. Adams, J. S. Fraser, EMRinger: side chain–directed model and map validation for 3D cryo-electron microscopy. *Nat Methods*. **12**, 943–946 (2015).

30. V. B. Chen, W. B. Arendall, J. J. Headd, D. A. Keedy, R. M. Immormino, G. J. Kapral, L. W. Murray, J. S. Richardson, D. C. Richardson, MolProbity: all-atom structure validation for macromolecular crystallography. *Acta Crystallogr D Biol Crystallogr*. **66**, 12–21 (2010).

31. F. Sievers, D. G. Higgins, Clustal Omega. *Current Protocols in Bioinformatics*. **48** (2014), doi:10.1002/0471250953.bi0313s48.

32. A. Hayer, L. Shao, M. Chung, L.-M. Joubert, H. W. Yang, F.-C. Tsai, A. Bisaria, E. Betzig, T. Meyer, Engulfed cadherin fingers are polarized junctional structures between collectively migrating endothelial cells. *Nat Cell Biol*. **18**, 1311–1323 (2016).

33. G. E. Peng, S. R. Wilson, O. D. Weiner, A pharmacological cocktail for arresting actin dynamics in living cells. *MBoC*. **22**, 3986–3994 (2011).
